# Critical domains for NACC2-NTRK2 fusion protein activation

**DOI:** 10.1371/journal.pone.0301730

**Published:** 2024-06-27

**Authors:** Wei Yang, April N. Meyer, Zian Jiang, Xuan Jiang, Daniel J. Donoghue

**Affiliations:** 1 Department of Chemistry and Biochemistry, University of California San Diego, La Jolla, California, United States of America; 2 UCSD Moores Cancer Center, University of California San Diego, La Jolla, California, United States of America; University of Florida College of Public Health & Health Professions, UNITED STATES

## Abstract

Neurotrophic receptor tyrosine kinases (NTRKs) belong to the receptor tyrosine kinase (RTK) family. NTRKs are responsible for the activation of multiple downstream signaling pathways that regulate cell growth, proliferation, differentiation, and apoptosis. NTRK-associated mutations often result in oncogenesis and lead to aberrant activation of downstream signaling pathways including MAPK, JAK/STAT, and PLCγ1. This study characterizes the NACC2-NTRK2 oncogenic fusion protein that leads to pilocytic astrocytoma and pediatric glioblastoma. This fusion joins the BTB domain (Broad-complex, Tramtrack, and Bric-a-brac) domain of NACC2 (Nucleus Accumbens-associated protein 2) with the transmembrane helix and tyrosine kinase domain of NTRK2. We focus on identifying critical domains for the biological activity of the fusion protein. Mutations were introduced in the charged pocket of the BTB domain or in the monomer core, based on a structural comparison of the NACC2 BTB domain with that of PLZF, another BTB-containing protein. Mutations were also introduced into the NTRK2-derived portion to allow comparison of two different breakpoints that have been clinically reported. We show that activation of the NTRK2 kinase domain relies on multimerization of the BTB domain in NACC2-NTRK2. Mutations which disrupt BTB-mediated multimerization significantly reduce kinase activity and downstream signaling. The ability of these mutations to abrogate biological activity suggests that BTB domain inhibition could be a potential treatment for NACC2-NTRK2-induced cancers. Removal of the transmembrane helix leads to enhanced stability of the fusion protein and increased activity of the NACC2-NTRK2 fusion, suggesting a mechanism for the oncogenicity of a distinct NACC2-NTRK2 isoform observed in pediatric glioblastoma.

## Introduction

NTRKs (Neurotrophic Receptor Tyrosine Kinases) represent a subfamily of receptor tyrosine kinase (RTK) proteins that are expressed in neuronal tissues [1]. The three members of the NTRK family, NTRK1, NTRK2, and NTRK3, all contain an extracellular ligand binding domain, a transmembrane domain, and an intracellular kinase domain [2]. Each NTRK is activated by its corresponding ligand [3], which upon binding to the extracellular domain, induces NTRK dimerization followed by rapid autophosphorylation of tyrosine residues in the intracellular kinase domain [4]. Once activated, NTRKs trigger multiple cell survival, proliferation, and apoptosis-related intracellular signaling pathways, including RAS/MAPK, PLCγ1, and JAK/STAT3 pathways [5]. NTRK point mutations or NTRK chromosomal translocation events often lead to aberrant activation of the kinase domain, resulting in carcinogenesis due to the constitutive activation of downstream signaling [6].

Since the discovery of the first NTRK fusion protein in 1982 [7], numerous studies have been conducted to discover the mechanisms for their oncogenic activation. Inhibitors have been developed against the NTRK kinase domain and have yielded promising therapeutic effects [8]. This study focuses on the NACC2-NTRK2 fusion protein, formed by fusion of NACC2 (Nucleus Accumbens-associated protein 2) with NTRK2. The first NACC2-NTRK2(ex4:ex13) fusion was discovered in pilocytic astrocytoma [9], which is a World Health Organization (WHO) grade 1 tumor exhibiting a 10-year survival rate over 90%. Later, another variant fusion (ex4:ex15) was discovered in pediatric glioblastoma [10], which is a rare WHO grade IV tumor. Throughout this work, if not noted explicitly, NACC2-NTRK2 refers to the more common NACC2-NTRK2(ex4:ex13) fusion, while NACC2-NTRK2(ex4:ex15) is always explicitly named.

NACC2 has two major functional domains: a BTB domain (Broad-complex, Tramtrack, and Bric-a-brac) that is responsible for NACC2 dimerization and recruitment of the NuRD (Nucleosome Remodeling and Deacetylase) transcriptional regulator complex to the MDM2 (Mouse Double Minute 2) promotor [11], and a BEN domain (named for “BANP, E5R and NACC1” where it was characterized), consisting of an alpha-helical module that mediates protein-DNA and protein-protein interactions during chromatin organization and transcription [12,13]. In the NACC2-NTRK2(ex4:ex13) fusion, residues 1–418 of NACC2 are fused with NTRK2. This sequence contains the BTB domain (residues 20–120), a disordered region (DR, residues 121–350), and a portion (residues 351–418) of the BEN domain.

The BTB domain is an evolutionarily conserved domain existing in various proteins, most often in zinc finger proteins serving as transcriptional regulators [14,15]. BTB domains are responsible for various protein-protein interactions, including self-association, hetero-multimerization, and transcription factor assembly [16]. The BTB domain contains multiple alpha helices which, upon dimerization, form a neatly intertwined dimer interface exhibiting a charged pocket of 4 charged residues in the center of the interface [17]. Disruption of the charged pocket interferes with dimer formation and results in a dysfunctional BTB domain [18]. In other BTB domain-containing fusion-positive cancers, such as PLZF/Retinoic Acid Receptor alpha (RAR alpha) that causes Acute Myeloid Leukemia (AML), disruption of the BTB domain also abrogates oligomer formation and fusion protein functions [19].

This study characterizes the function of each domain of the NACC2-NTRK2 fusion protein and its contribution to the aberrant activation of the fusion. We demonstrate that the fusion protein activates the downstream signaling pathways of NTRK2 independently of the ligand BDNF (Brain-Derived Neurotrophic Factor). Disruption of the BTB domain, whether by mutation in the charged pocket or the monomer core, leads to a reduction in multimer formation and abrogates the NIH3T3 transformation activity of the fusion protein. To investigate the function of the retained portion of NACC2 downstream of the BTB domain, which includes the disordered region (residues 121–350) and a portion of the BEN domain (residues 351–418), we constructed a new fusion that contains only the NACC2 BTB domain (residue 1–120) and the NTRK2 tyrosine kinase domain. Results show that this region, the disordered region and partial BEN domain, provides covalent linkage for the NACC2-NTRK2 multimer. Removal of the region between the BTB domain and the NTRK2 fusion point results in reduced downstream activation and decreased biological activity.

Furthermore, we compared the (ex4:ex13) fusion identified in pilocytic astrocytoma with the (ex4:ex15) fusion identified in pediatric glioblastoma, which differ by the presence or absence of NTRK2 exons 13 and 14 that encode the TM (transmembrane) helix. Removal of either the TM helix alone, or the complete removal of exons 13 and14, results in a significant increase in protein stability and biological activity. These data suggest that a degron exists within the sequence of the TM helix and that the (ex4:ex15) fusion protein, lacking this sequence, exhibits greater stability and biological activity.

## Results

### NACC2-NTRK2 promotes ligand-independent kinase and downstream activation

**Fig 1:**
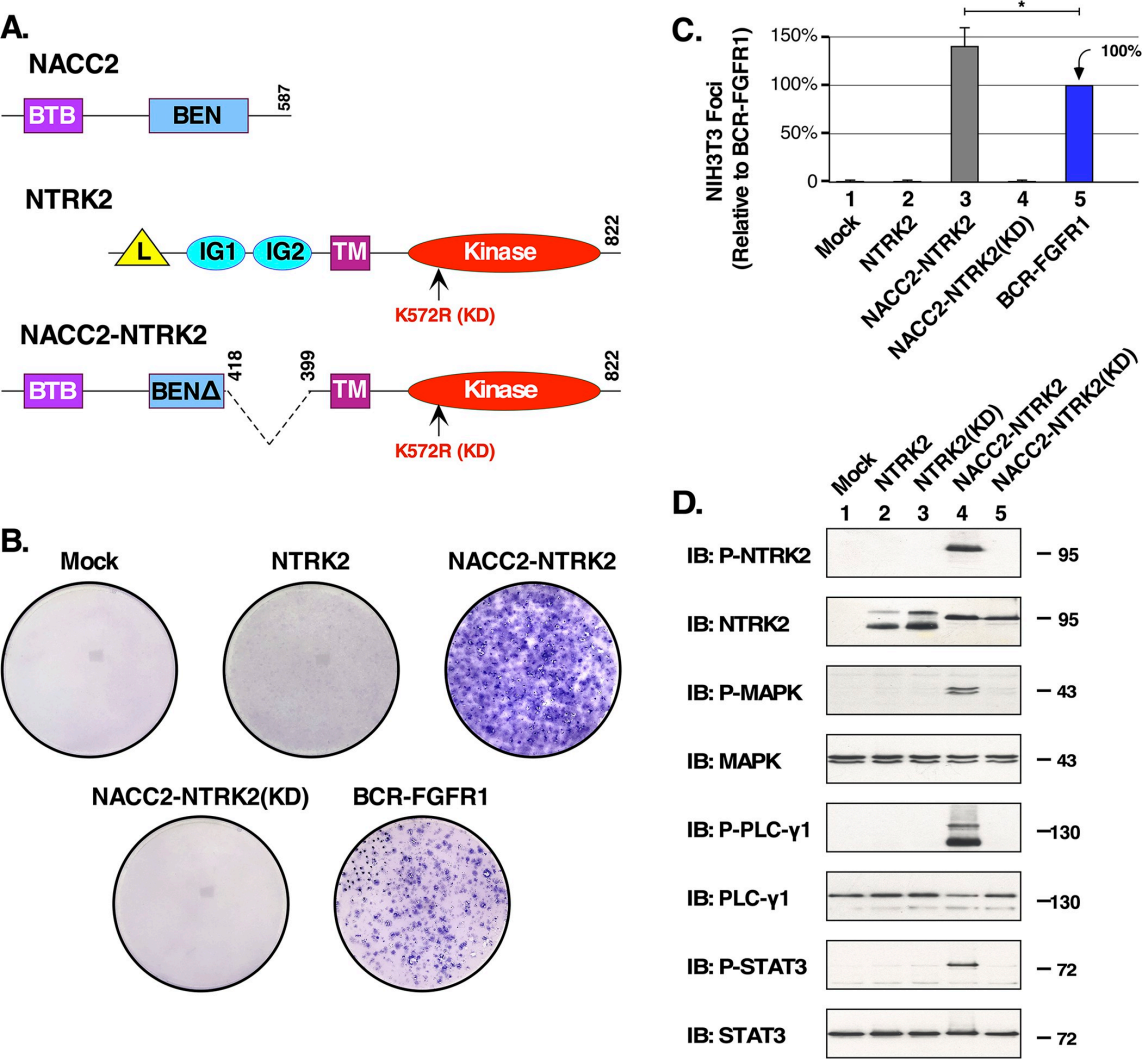
NACC2-NTRK2 exhibits biological activity in cell transformation and cell signaling assays. **(A)** Schematic presents NACC2, NTRK2, and NACC2-NTRK2 fusion with K572R kinase dead mutation (KD). “L” refers to Leucine-rich domain, “IG1” and “IG2” refer to Immunoglobulin-like domains 1 and 2, “BTB” refers to Broad-Complex, Tramtrack and Bric a brac domain, “BEN” refers to an adapter domain found in BANP, E5R, and NACC1 proteins, “TM” refers to the Transmembrane helix, and “Kinase” refers to the tyrosine kinase domain of NTRK3. The kinase dead (KD) mutation K572R is also shown in NTRK2 and NACC2-NTRK2. Placement of domains is only approximate. **(B)** Representative plates of NIH3T3 cell transformation assays are shown for NTRK2, NACC2-NTRK2, and NACC2-NTRK2(KD). BCR-FGFR1 is included as positive control. In this experiment, all constructs were assayed in three independent replicates, except for NACC2-NTRK2 which was assayed six times, and BCR-FGFR1 which was assayed five times. **(C)** Results of NIH3T3 focus assays are presented. Each construct was assayed a minimum of three times, and the ratio of foci/G418-resistant colonies was calculated as a percentage of transformation +/- SEM relative to BCR-FGFR1. The *P* value of a two-tailed paired t test comparing NACC2-NTRK2 with BCR-FGFR1 was 0.011, and is shown as * = *P* ≤0.05. **(D)** The activation of downstream signaling pathways is presented. HEK293T cell lysates expressing NTRK2, NTRK2(KD), NACC2-NTRK2, and NACC2-NTRK2(KD) were analyzed by SDS-PAGE and immunoblotted for P-NTRK (top panel), total NTRK2 (2^nd^ panel), P-MAPK (3^nd^ panel), total MAPK (4^th^ panel), P-PLCγ1 (5th panel, upper band), total PLCγ1(6^th^ panel), P-STAT3(7^th^ panel) and total STAT3(8^th^ panel). Note that in this figure, and other immunoblots of P-PLCγ1, the band of interest migrates above the 130 kD marker; the prominent band near the bottom of this blot represents antiserum cross-reactivity with the activated NTRK2 kinase domain.

NACC2 consists of 587 residues (Uniprot Q96BF6) encoded by 5 exons, whereas NTRK2 contains 822 residues (Uniprot Q16620) encoded by 21 exons. NACC2-NTRK2 contains the NACC2 BTB dimerization domain and partial BEN domain, the NTRK2 transmembrane helix, and the NTRK2 tyrosine kinase domain (Fig 1A). The normal activation of the NTRK2 kinase relies on dimerization, which is induced by BDNF ligand binding to the extracellular domain. To examine the transforming activity of the NTRK2 kinase domain, in the context of the fusion protein NACC2-NTRK2, focus-forming assays were preformed in NIH3T3 cells. Transformation assays represent one of the original assays used to identify and characterize oncogenes [20–23]. Transfected NIH3T3 cells expressing an oncogene are able to overgrow the contact-inhibited monolayer of the cells and establish foci. The expression and efficiency of the transfections are determined by plating an aliquot of the transfected cells into selection media containing Geneticin. The colonies that form are then counted and used to quantitate the focus-forming ability of each clone. We included BCR-FGFR1 fusion protein, previously described, as a positive control [24]. Using this assay, expression of wild-type NACC2, NTRK2, and NACC2-NTRK2(KD) showed no focus-forming activity, whereas the NACC2-NTRK2 fusion showed high transformation activity, indicating that an active NTRK2 kinase domain is essential for the cell transforming ability of NACC2-NTRK2 fusion proteins (Fig 1B and 1C).

Next, we examined the downstream signaling activity that results from NACC2-NTRK2 activation. NTRK2, NTRK2(KD), NACC2-NTRK2, and NACC2-NTRK2(KD) were transfected into HEK293T cells, previously utilized to characterize downstream signaling stimulated by the oncogenic fusion protein FGFR3-TACC3 [25]. NTRK2(KD) was included as a negative control. We analyzed activation of MAPK, PLCγ1, and JAK/STAT pathways, shown previously to be activated in response to NTRK2 signaling [26]. Cells were collected and lysed in RIPA, and then analyzed by SDS-PAGE and immunoblotting with appropriate antibodies. Activation of downstream pathways was only observed in lysates expressing NACC2-NTRK2, as shown by immunoblotting for P-NTRK2, P-MAPK, P-PLCγ1, and P-STAT3 (Fig 1D, lane 4, 1^st^, 3^rd^, 5^th^, and 7^th^ panels). In contrast, no activation of these pathways was observed in cells expressing NTRK2, NTRK2(KD), of NACC2-NTRK2(KD). Importantly, the immunoblot for total NTRK2 (Fig 1D, 2^nd^ panel) shows approximately equivalent expression of NTRK2 and the NACC2-NTRK2 fusion protein, both wild type and KD. Together, these data demonstrate that a functional tyrosine kinase domain in NACC2-NTRK2 is required for activation of downstream signaling pathways and transformation of NIH3T3 cells.

### The NACC2 BTB domain is responsible for multimerization

**Fig 2:**
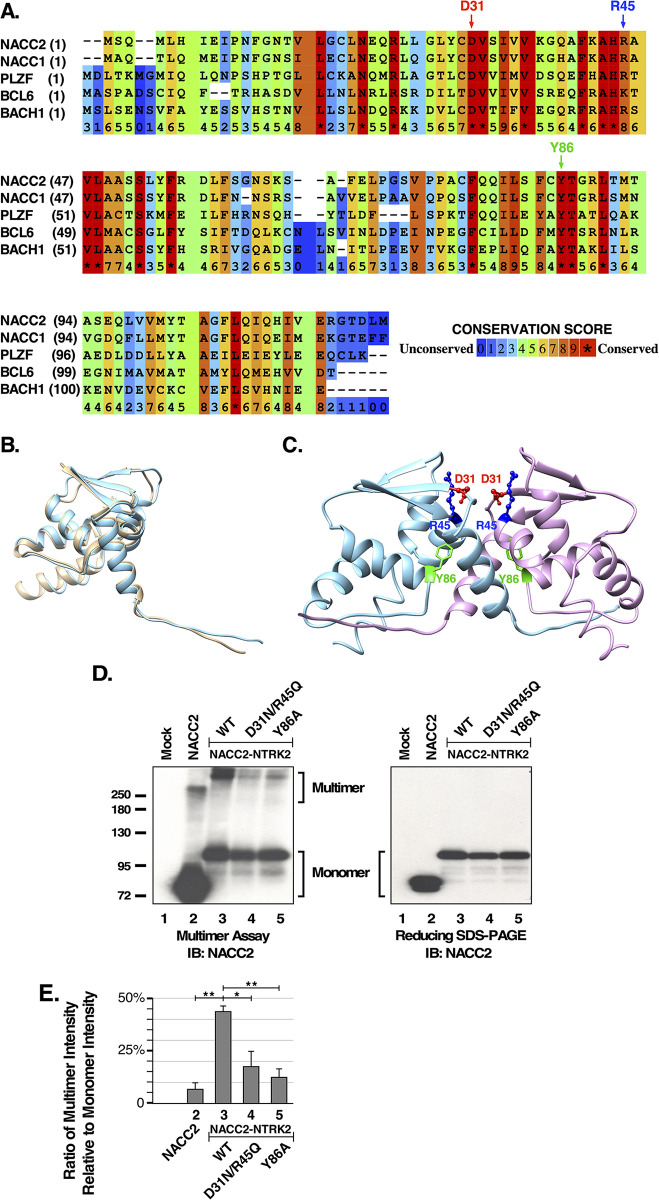
The BTB domain of NACC2 is responsible for multimerization of NACC2-NTRK2. **(A)** The sequence alignment is presented of the NACC2 BTB domain in comparison with four other BTB domains of proteins that have been structurally determined. The sequences shown include human NACC2 (UniProt Q96BF6), human NACC1 (UniProt Q96RE7), human PLZF (UniProt Q05516), human BCL6 (UniProt P41182), and human BACH1 (UniProt O148667). This alignment reveals several conserved residues shown by others to be critical for PLZF dimerization, including the D31/R45 charged pocket residues and Y86 monomer core residue [17,18]. Multi-sequence alignment was performed using Praline [29]. **(B)** The structural alignment is presented showing the BTB domains of NACC2 (AlphaFold: AF-Q9DCM7-F1, blue) and PLZF (PDB: 1BUO, tan), created using Chimera. **(C)** A model of the NACC2 BTB domain dimer structure was created based on the PLZF dimer (PDB ID: 1BUO). The charged pocket residues D31 and R45 are indicated at the dimer interface, and the monomer core residue Y86 is also shown. **(D)** Lysates from HEK293T cells expressing NACC2, NACC2-NTRK2, NACC2(D31N/R45Q)-NTRK2, and NACC2(Y86A)-NTRK2 were analyzed by SDS-PAGE and immunoblotted for NACC2. Non-reducing sample buffer (left panel) or reducing sample buffer (right panel) was used for duplicate samples. Significant multimer bands of NACC2 and NACC2-NTRK2 were observed in the non-reducing conditions (left panel, lanes 2 and 3). **(E)** The ratio of Multimer intensity relative to Monomer intensity is shown, using intensities of three replicates quantitated by ImageJ. The P values of two-tailed paired t tests are shown: For NACC2-NTRK2 versus NACC2, P = 0.0020, indicated as ** = P ≤0.01; for NACC2-NTRK2 versus NACC2(D31N/R45Q)-NTRK2, P = 0.023, indicated as * = P ≤0.05; and for NACC2-NTRK2 versus NACC2(Y86A)-NTRK2, P = 0.0023, indicated as ** = P ≤0.01.

After confirming the oncogenic activation of the NACC2-NTRK2 fusion protein, we tried to elucidate the function of the N-terminal fusion partner NACC2. In the fusion, the entire BTB domain (residue 20–120), part of the BEN domain (residue 351–418), and a large disordered region between these two domains are retained. We endeavored to characterize the relationship between the BTB domain, protein multimerization, and activation of the associated tyrosine kinase domain. We also wished to identify important regulatory residues whose disruption would result in inactivation of the fusion. Currently, there exists no detailed structure-function analysis of the NACC2 BTB domain. However, the BTB domain is an evolutionarily conserved domain retained by various protein families in different species [27]. We performed multisequence alignment of the NACC2 BTB domain with four other BTB domains that have all been structurally determined: NACC1, PLZF, BCL6, and BACH1 (Fig 2A). Multisequence alignment indicated that several conserved motifs that have been identified in BTB proteins such as PLZF and BCL6, also exist in NACC2. These motifs include a charged pocket motif that consists of a negatively charged aspartic acid (D) together with a positively charged arginine (R) or lysine (K), and a monomer core motif that consists of a tyrosine residue. These two motifs exist in all five BTB domain proteins presented (Fig 2A). Using Chimera, we compared the PLZF BTB domain structure (PDB ID: 1BUO) with the NACC2 BTB domain predicted by AlphaFold (AF-Q9DCM7-F1) [17,28]. The PLZF BTB domain and NACC2 BTB domain are highly similar in both structure and sequence (Fig 2A and 2B); thus, due to the conservation of BTB domains, mutations in conserved residues that disrupt multimerization in PLZF and BCL6 [17] could potentially disrupt multimerization of the NACC2 BTB domain (Fig 2C). Based on this comparison, we introduced charged pocket (D31N R45Q) and monomer core (Y86A) mutations into the BTB domain of the NACC2-NTRK2 fusion protein and examined their effects on multimerization.

The BTB domain mutants were expressed in HEK293T cells, collected, and lysed in E1A lysis buffer to stabilize protein-protein interactions [19,30]. Samples were prepared in non-reducing sample buffer and resolved by SDS-PAGE followed by immunoblotting. Using this protocol, high molecular weight multimeric complexes were observed (Fig 2D, left panel, lane 3). The approximate molecular weight of the observed multimeric complexes suggests that NACC2-NTRK2 primarily forms a quaternary structure. Incorporation of either the D31N/R45Q or Y86A mutations resulted in a significant reduction in homo-multimer formation (Fig 2D, left panel, lanes 4–5). Quantitation of the ratio of the multimer band compared to the monomer band (Fig 2E) indicated a significant reduction from 44% for the wild-type NACC2-NTRK2 fusion to 18% due to the charged pocket mutations D31N/R45Q, and a reduction from 44% to 12% due to the monomer core mutation Y86A. Surprisingly, the data indicate that all of the NACC2-NTRK2 fusion proteins multimerize more efficiently than NACC2 alone, which exhibited a multimer/monomer ratio of only 7%.

When these same samples were prepared with sample buffer containing β-mercaptoethanol to reduce disulfide bonds, multimeric bands were reduced to monomeric proteins (Fig 2D, right panel). This suggests the existence of covalent interactions stabilizing NACC2-NTRK2 multimerization.

**Fig 3:**
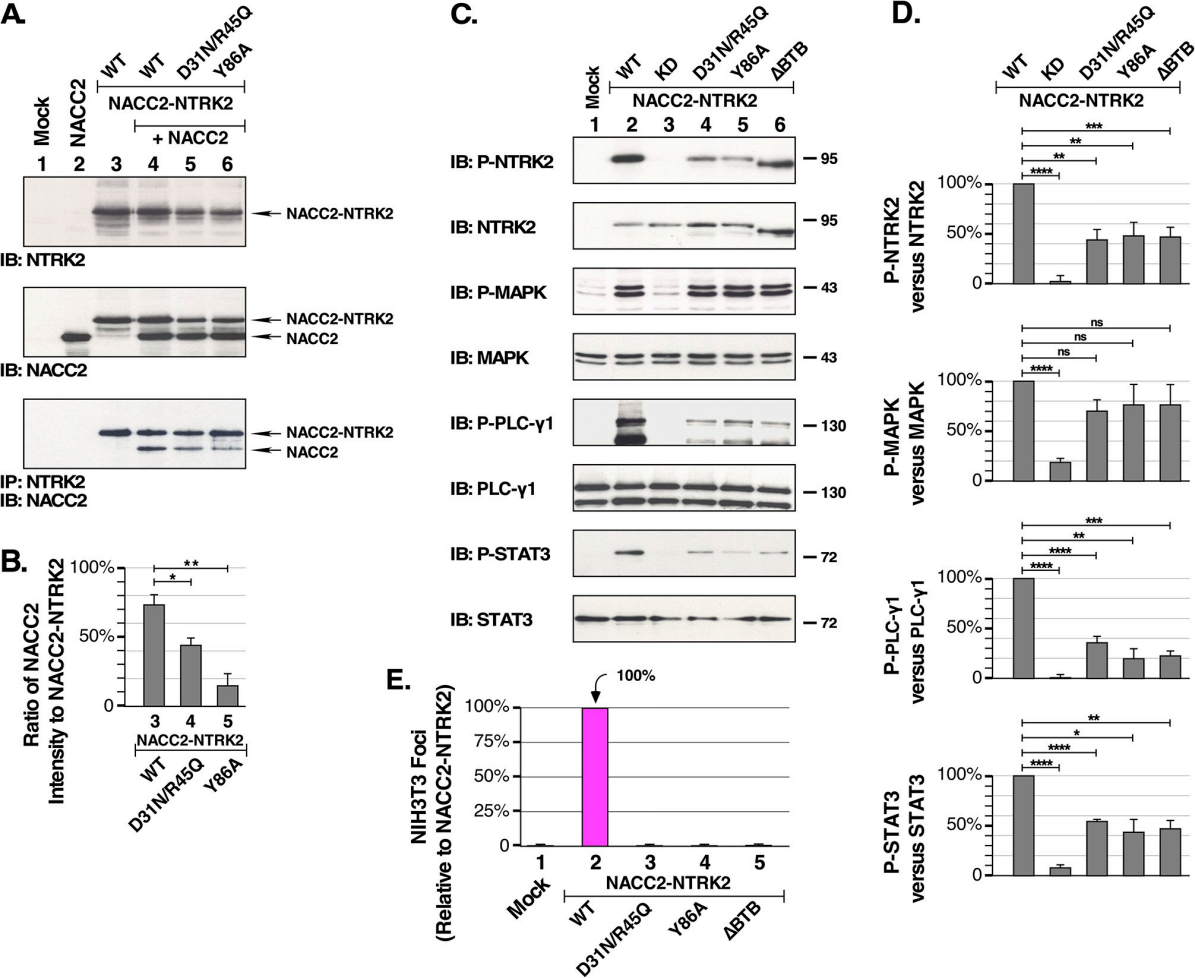
The BTB domain of NACC2 is required for biological activity of NACC2-NTRK2. **(A)** The interaction of NACC2 and NACC2-NTRK2 is examined. Lysates of HEK293T cells expressing NACC2-NTRK2 (designated as WT), or with the mutations D31N/R45Q or Y86A, were coexpressed with NACC2 as indicated (Lanes 4–6). Samples were prepared in E1A buffer to preserve protein-protein interactions. The 3^rd^ panel presents the results of immunoprecipitation using antibodies against NTRK2, to recover the NACC2-NTRK fusion proteins, followed by immunoblotting to detect NACC2 present in the immune complexes. Lane 4 clearly shows binding of NACC2 to NACC2-NTRK2, which is reduced in the presence of the mutations D31N/R45Q (lane 5), or Y86A (lane 6). Control immunoblots are shown for NTRK2 (top panel) and NACC2 (middle panel). (B) The ratio of the intensity of NACC2 relative to the NACC2-NTRK2 fusion with which it was co-immunoprecipitated is shown, using intensities of multiple replicates quantitated by ImageJ. The P values of two-tailed paired t tests are shown: For NACC2 bound to NACC2-NTRK2 versus NACC2(D31N/R45Q)-NTRK2, P = 0.034, indicated as * = P ≤0.05; for NACC2 bound to NACC2-NTRK2 versus NACC2(Y86A)-NTRK2, P = 0.003, indicated as ** = P ≤0.01. **(C)** HEK293T cells expressing NACC2-NTRK2, NACC2-NTRK2(KD), NACC2(D31N/R45Q)-NTRK2, NACC2(Y86A)-NTRK2 or NACC2(ΔBTB)-NTRK2 were examined for the activation of downstream signaling pathways. Cell lysates were analyzed by SDS-PAGE and immunoblotted for P-NTRK (top panel), total NTRK2 (2^nd^ panel), P-MAPK (3^nd^ panel), total MAPK (4^th^ panel), P-PLCγ1 (5th panel, upper band), total PLCγ1 (6^th^ panel), P-STAT3(7^th^ panel) and total STAT3(8^th^ panel). **(D)** The immunoblots presented in (C), together with additional independent replicates, were quantitated by ImageJ and used to calculate the changes in P-NTRK2, P-MAPK, P-PLCγ1, and P-STAT3, after normalization of each sample in comparison to total NTRK2, MAPK, PLCγ1, and STAT3. The P values of two-tailed paired t tests are shown for wild-type NACC2-NTRK2 versus each of the mutants KD, D31N/R45Q, Y86A, and ΔBTB. Statistical significance is indicated as follows: ns = not significant; * = P ≤0.05; ** = P ≤0.01; *** = P ≤0.001; **** = P ≤0.0001. Four independent replicates were used to calculate the changes in P-NTRK2 relative to NTRK2, and three independent replicates were used for changes in P-MAPK, P-PLCγ1, and P-STAT3. **(E)** The BTB domain mutants of NACC2-NTRK2 were examined for NIH3T3 cell transformation activity. In this experiment, all constructs were assayed in three independent replicates, except for NACC2-NTRK2 which was assayed six times. The ratio of foci/G418-resistant colonies was calculated as a percentage of transformation +/- SEM relative to NACC2-NTRK2, which was set to 100%.

In addition, we employed an assay for hetero-multimerization. The BTB domain mutants were expressed either alone or in combination with wild-type NACC2 in HEK293T cells (Fig 3A). Lysates were immunoprecipitated using antibodies against NTRK2, after which these immune complexes were analyzed by SDS-PAGE and immunoblotting using antibodies against NACC2. We postulated that the BTB domain present in NACC2-NTRK2 would undergo multimerization with the BTB domain of wild-type NACC2. As anticipated, NACC2-NTRK2 was observed to bind NACC2 (Fig 3A, bottom panel, lane 4), while incorporation of either the D31N/R45Q or Y86A mutants resulted in diminished binding of NACC2 (Fig 3A, bottom panel, lanes 5 and 6*)*. Using the bottom panel of Fig 3A together with similar replicates, quantitation of the ratio NACC2 relative to the NACC2-NTRK2 fusion with which it was co-immunoprecipitated was determined (Fig 3B). This revealed a significant reduction from 72% for the wild-type NACC2-NTRK2 fusion to 45% due to the charged pocket mutations D31N/R45Q, and a reduction from 72% to 15% due to the monomer core mutation Y86A. These changes were statistically significant when compared in a two-tailed paired t test. These data indicate that introduction of either the charged pocket mutations or the monomer core mutation into NACC2-NTRK2 resulted in a significant reduction in its ability to bind wild type NACC2 in this hetero-multimerization assay.

Taken together, the assays examining multimerization of NACC2-NTRK2 and hetero-multimerization with NACC2 both demonstrate the importance of the BTB domain in the formation of these complexes. Both assays also demonstrate the ability of mutations within the BTB domain, such as the charged pocket (D31N/R45Q) and monomer core (Y86A) mutations, to disrupt multimerization.

### NACC2-NTRK2 fusion activity requires BTB domain multimerization

The extracellular ligand BDNF induces dimerization of NTRK2, resulting in auto-phosphorylation of the NTRK2 kinase domain. In an oncogenic fusion protein such as NACC2-NTRK2, ligand-mediated activation is impossible due to deletion of the extracellular ligand-binding domain. This necessitates dimerization by another mechanism, usually provided by the N-terminal fusion partner [31,32]. We next examined the importance of a functional BTB domain in NACC2-NTRK2 for activation of downstream signaling pathways, as indicated by phosphorylation of MAPK, PLCγ1, or STAT3. In addition to the charged pocket (D31N/R45Q) and monomer core (Y86A) mutations discussed above, we also included an additional mutation, ΔBTB, which deletes the entire BTB domain. Results show that the specific BTB point mutations D31N/R45Q and Y86A were able to reduce PLCγ1 and STAT-3 activation to the same extent as deletion of the entire BTB domain itself (Fig 3C, 5^th^ and 7^th^ panels). All three BTB domain mutations were observed to partially reduce receptor autophosphorylation, as detected by immunoblotting for phospho-NTRK2 (Fig 3C, top panel). Somewhat surprisingly, the BTB domain mutations did not appear to significantly reduce MAPK activation, which remained similar to the wild-type NACC2-NTRK2 fusion (Fig 3C, 3^rd^ panel). Importantly, the immunoblot for total NTRK2 (Fig 3C, 2^nd^ panel) shows approximately equivalent expression of the NACC2-NTRK2 fusion proteins examined here.

Using multiple replicates of immunoblots, the relative changes were quantitated as shown in Fig 3D. For all four phospho-proteins examined, P-NTRK2, P-MAPK, P-PLCγ1, and P-STAT3, the difference between wild-type NACC2-NTRK2 versus the KD derivative was statistically significant. For P-MAPK activation, the differences between wild-type NACC2-NTRK2 and the mutants D31N/R45Q, Y86A, and ΔBTB were not significant, suggesting that robust MAPK activation can be achieved by a NACC2-NTRK2 mutant which is inactive in other assays, but which retains an intact tyrosine protein kinase domain. However, for the activation of P-PLCγ1 and P-STAT3, the loss of activity exhibited by the D31N/R45Q, Y86A, and ΔBTB mutants was statistically significant in every case as shown in Fig 3D.

When examined for biological activity using NIH3T3 cell transformation, all three BTB domain mutants were completely inactive in their ability to produce foci of transformed cells (Fig 3E).

Collectively, these data demonstrate that disruption of oligomerization mediated by the BTB domain, either by specific point mutation or by deletion, results in inactivation of the NACC2-NTRK2 fusion protein as evidenced by decreased activation of downstream PLCγ1 and STAT-3 signaling pathways and by loss of biological activity in a morphological transformation assay. The continued activation of MAPK signaling activity by these same BTB domain mutations suggests that activation of this pathway alone is insufficient to support the biological activity of NACC2-NTRK2.

### NACC2 residues 121–418 stabilize NACC2-NTRK2 multimerization via covalent interactions

**Fig 4:**
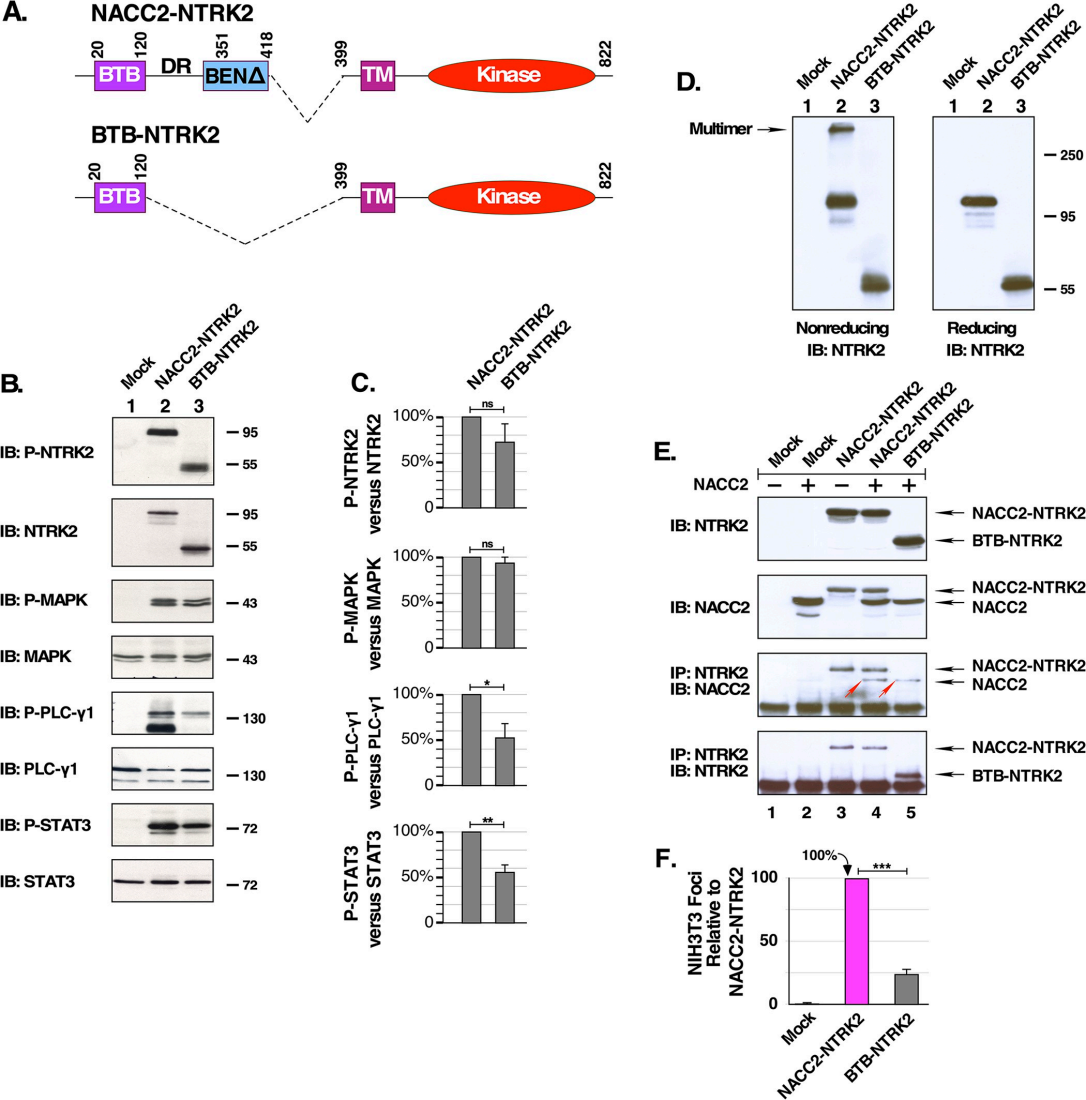
NACC2 residues 121–418 provides potential covalent stabilization for NACC2-NTRK2 multimer. **(A)** NACC2-NTRK2 is shown schematically with the BTB domain from residues 20–120, a partial BEN domain at residues 351–418, and the disorder region (DR) in between. Also shown in comparison is the deletion of residues 120–418 of NACC2, which retains only the N-terminal BTB domain, designated BTB-NTRK2. **(B)** Downstream activation by BTB-NTRK2. HEK293T cells expressing NACC2-NTRK2 or BTB-NTRK2 were examined for activation of downstream signaling pathways, as described previously for Figs 1D and 3C. **(C)** The immunoblots presented in (B), together with additional independent replicates, were quantitated by ImageJ and used to calculate the changes in P-NTRK2, P-MAPK, P-PLCγ1, and P-STAT3, after normalization of each sample in comparison to total NTRK2, MAPK, PLCγ1, and STAT3. The P values of two-tailed paired t tests are shown for wild-type NACC2-NTRK2 versus BTB-NTRK2. Statistical significance is indicated as follows: ns = not significant; * = P ≤0.05; ** = P ≤0.01. Three independent replicates were used for each condition, except for P-STAT3 for which five independent replicates were available. **(D)** Lysates of HEK293T cells expressing NACC2-NTRK2 or BTB-NTRK2 were examined for homo-multimerization. Samples were loaded using non-reducing sample buffer (left panel), or reducing sample buffer containing β-mercaptoethanol (right panel), and then analyzed by SDS-PAGE and immunoblotted for NTRK2. **(E)** Hetero-multimerization assay of NACC2-NTRK2 and BTB-NTRK2. Fusion clones were cotransfected with NACC2 (lanes 4 and 5) and examined for their ability to bind to NACC2 in an immune complex prepared using antibodies against NTRK2. The 3^rd^ panel shows clearly the binding of NACC2 to NACC2-NTRK2 (lane 4), indicated by a red arrowhead. NACC2 also clearly binds to BTB-NTRK2 (lane 5), again indicated by a red arrowhead. Control lysate blots are shown for NTRK2 (1^st^ panel) and NACC2 (2^nd^ panel). Control IP immunoblot is shown in 4^th^ panel for NTRK2. **(F)** The BTB-NTRK2 fusion was compared with NACC2-NTRK2 in NIH3T3 cells transformation assays. In this experiment, NACC2-NTRK2 was assayed in six independent replicates, and BTB-NTRK2 was assayed three times. The ratio of foci/G418-resistant colonies was calculated as a percentage of transformation +/- SEM relative to NACC2-NTRK2, which was set to 100%. The P value of a two-tailed paired t test comparing NACC2-NTRK2 with BTB-NTRK2 was 0.00091, and is shown as *** = P ≤0.001.

To investigate the possible role of the retained portion of NACC2 downstream of the BTB domain, which includes the disordered region (residues 121–350) and the beginning of the BEN domain (residues 351–418), we constructed a shorter NACC2:NTRK2 fusion protein that contains only the BTB domain, residues 1–120, referred to as BTB-NTRK2 (Fig 4A). We examined activation of downstream signaling pathways, protein multimerization, and biological activity for BTB-NTRK2 in comparison with NACC2-NTRK2. This fusion protein results in similar levels of NTRK2 autophosphorylation, as well as similar levels of MAPK activation (Fig 4B, 1^st^ and 3^rd^ panels). However, phosphorylation of PLCγ1 and STAT3 by BTB-NTRK2 was diminished relative to NTRK2-NACC2 (Fig 4B, 5^th^ and 7^th^ panels). Importantly, the immunoblot for total NTRK2 (Fig 4B, 2nd panel) shows approximately equivalent expression of NACC2-NTRK2 in comparison with BTB-NTRK2. Quantitation of replicate immunoblots (Fig 4C) revealed that BTB-NTRK2 exhibited significantly decreased phosphorylation of PLCγ1 and STAT3, although no significant change was observed in P-NTRK2 or P-MAPK.

We next examined the protein multimerization of BTB-NTRK2. As described above, samples were prepared in non-reducing sample buffer and resolved by SDS-PAGE to examine multimerization. As before, NACC2-NTRK2 exhibited significant multimerization (Fig 4D, lane 2); in contrast, BTB-NTRK2 did not exhibit detectable multimerization (Fig 4D, lane 3). This assay demonstrates that the NACC2-NTRK2 multimer is partially resistant to SDS denaturation under non-reducing conditions, whereas a putative BTB-NTRK2 multimer was not detectable under these conditions.

In the hetero-multimerization assay, BTB-NTRK2 was examined for its ability to bind wild-type NACC2 when co-expressed. Cell lysates were immunoprecipitated using antibodies against NTRK2, after which they were analyzed by SDS-PAGE and immunoblotting with antibodies against NACC2. Binding of NACC2 was observed with both NACC2-NTRK2 and with BTB-NTRK2 (Fig 4E, panel 3, lanes 4 and 5), and the location of the co-immunoprecipitated NACC2 is indicated by the red arrowheads.

When biological activity was examined by NIH3T3 transformation assay, BTB-NTRK2 exhibited only 25% transformation activity relative to NACC2-NTRK2 (Fig 4F). Considered together, the results of the multimerization assay and the hetero-oligomerization assay, together with the transformation assay, indicate that the BTB domain alone is sufficient for oncogenic activation, but residues 121–418 apparently contribute to the covalent stabilization and greater activation of NACC2-NTRK2 compared with BTB-NTRK2.

### The NTRK2 transmembrane helix contains a putative degron

**Fig 5:**
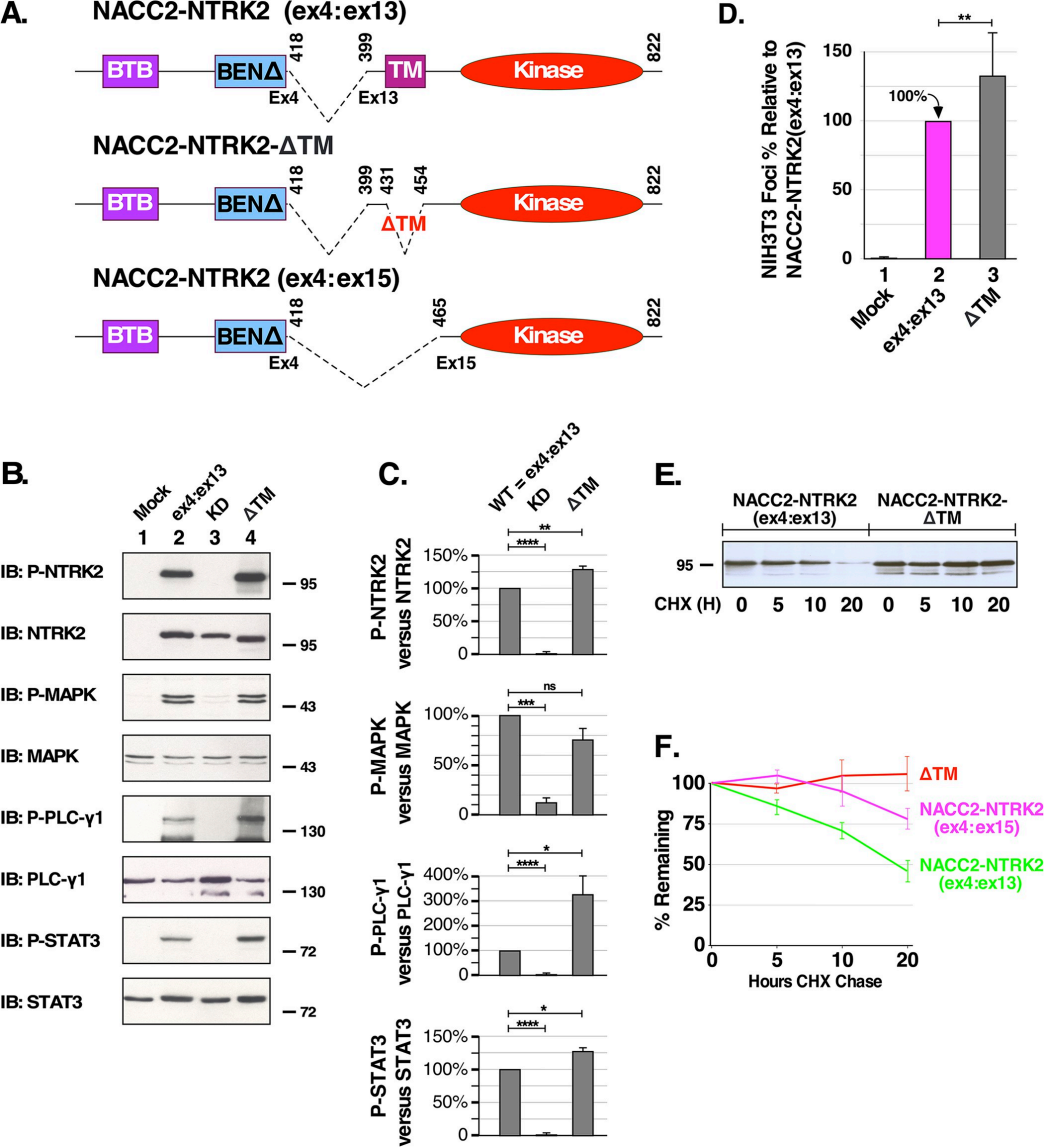
Deletion of the transmembrane helix promotes greater stability and biological activity. **(A)** NACC2-NTRK2 examined thus far in this work is shown as NACC2-NTRK2(ex4:ex13), in comparison with two derivatives: NACC2-NTRK2-ΔTM, deleting just the transmembrane helix, and NACC2-NTRK2(ex4:ex15) which deletes NTRK2 exons 13 and 14. **(B)** HEK293T cells expressing NACC2-NTRK2, NACC2-NTRK2-ΔTM and NACC2-NTRK2(ex4:ex15) were examined for activation of downstream signaling pathways, as described previously for Figs 1D and 3C. **(C)** The immunoblots presented in (B), together with additional independent replicates, were quantitated by ImageJ and used to calculate the changes in P-NTRK2, P-MAPK, P-PLCγ1, and P-STAT3, after normalization of each sample in comparison to total NTRK2, MAPK, PLCγ1, and STAT3. The P values of two-tailed paired t tests are shown for wild-type NACC2-NTRK2 versus each of the mutants KD and ΔTM. Statistical significance is indicated as follows: ns = not significant; * = P ≤0.05; ** = P ≤0.01; *** = P ≤0.001; **** = P ≤0.0001. Three independent replicates were used for each condition. **(D)** The biological activity of NACC2-NTRK2(ex4:ex13) was compared to NACC2-NTRK2-ΔTM using NIH3T3 cell transformation assays. In this experiment, both NACC2-NTRK2(ex4:ex13) and NACC2-NTRK2-ΔTM were assayed in six independent replicates. The ratio of foci/G418-resistant colonies was calculated as a percentage of transformation +/- SEM relative to NACC2-NTRK2(ex4:ex13), which was set to 100%. The P value of a two-tailed paired t test comparing NACC2-NTRK2(ex4:ex13) with NACC2-NTRK2-ΔTM was 0.0096, and is shown as ** = P ≤0.01. **(E)** Lysates prepared from cells expressing either NACC2-NTRK2 (left lanes) or NACC2-NTRK2-ΔTM (right lanes) and treated with cycloheximide for the indicated time points were examined by SDS-PAGE and immunoblotted for NTRK2 The left lanes show decreasing amounts of NACC2-NTRK2 in the presence of cycloheximide, while the right lanes reveal little or no change in NACC2-NTRK2-ΔTM under identical conditions. **(F)** The half-life of NACC2-NTRK2(ex4:13) was determined to be approximately 18 hours, that of NACC2-NTRK2(ex4:15) approximately 42 hours, while the half-life of NACC2-NTRK2-ΔTM could not be determined due to its stability over the time course of this experiment. Quantitation was accomplished using ImageJ with a minimum of three replicates for each sample, and half-lives were determined by least squares analysis.

The NACC2-NTRK2 fusion utilized thus far throughout the present work, with exon 4 of NACC2 joined to exon 13 of NTRK2, is known to cause pilocytic astrocytoma. Going forward, we will refer to this fusion as NACC2-NTRK2(ex4:ex13) (Fig 5A, top). However, an isoform of NACC2-NTRK2 exhibiting a different breakpoint, NACC2-NTRK2(ex4:ex15) (Fig 5A, bottom), was observed in a case of pediatric glioblastoma [10]. We wished to investigate possible differences between these two NACC2-NTRK2 fusions that might explain the more severe pediatric glioblastoma resulting from the exon4:exon15 fusion. The most notable difference between these two fusions is the presence or absence of the TM helix. During the normal translation of NTRK2, an N-terminal signal peptide directs co-translational membrane insertion of the nascent polypeptide, a process which is then arrested upon membrane insertion of the TM domain to produce a Type I transmembrane protein. Lacking an N-terminal signal peptide, due to the substitution of the N-terminal domain by NACC2, either fusion of NACC2-NTRK2 is incapable of membrane insertion; consequently, the normal function of the TM helix as a membrane anchor is abrogated.

To approach this experimentally, we first deleted residues 431–454 of NACC2-NTRK2(ex4:ex13) to create NACC2-NTRK2-ΔTM (Fig 5A, middle); this deletes residues that encode the TM helix. This was first assayed for its downstream signaling activity and transformation ability in comparison with NACC2-NTRK2(ex4:ex13). Results show that NACC2-NTRK2-ΔTM increases NTRK2 autophosphorylation, as well as increasing PLCγ1 and STAT3 phosphorylation (Fig 5B, 1^st^, 5^th^, and 7^th^ panels). Importantly, the immunoblot for total NTRK2 (Fig 5B, 2nd panel) shows approximately equivalent expression of the mutants examined here: NACC2-NTRK2(ex4:ex15), NACC2-NTRK2(KD), and NACC2-NTRK2-ΔTM. Quantitation of replicate immunoblots (Fig 5C) revealed that NACC2-NTRK2-ΔTM exhibited significantly greater phosphorylation of P-NTRK2, P-PLCγ1 and P-STAT3, although no significant change was observed in P-MAPK. When assayed for biological activity using an NIH3T3 transformation assay, NACC2-NTRK2-ΔTM exhibited approximately 125% greater transformation activity compared with NACC2-NTRK2(ex4:ex15) (Fig 5D). A Student’s t-test of these data returned a p-value of 0.09, indicating the significance of this difference.

Next, cycloheximide chase experiments were utilized to compare the stability of NACC2-NTRK2-ΔTM and NACC2-NTRK2(ex4:ex13). Because cycloheximide blocks protein translation, the amount of protein remaining at different times reflects the half-life of the protein. HEK293T cells expressing the fusion proteins were treated with 300 ng/ml of cycloheximide for 0, 5, 10, and 20 hours before preparing cell lysates, analysis by SDS-PAGE, and immunoblotting. After addition of cycloheximide, the expression level of NACC2-NTRK2(ex4:ex13) decreased over time (Fig 5E, left lanes). In marked contrast, the observed amount of NACC2-NTRK2-ΔTM remained stable over the course of the experiment with little or no apparent degradation (Fig 5E, right lanes). When this was quantitated (Fig 5F), the half-life of NACC2-NTRK2 was determined to be approximately 18 hours, whereas the greater stability of NACC2-NTRK2-ΔTM precluded calculation of its half-life over the time course of this experiment.

As presented in Fig 5A, we also constructed a more extensive deletion in which all of exons 13 and 14 of NTRK2 were removed, to create the NACC2-NTRK2(ex4:ex15) fusion. When the stability of NACC2-NTRK2(ex4:ex15) was examined, the half-life was determined to be approximately 42 hours, more than twice that of the more commonly observed NACC2-NTRK2(ex4:ex13) fusion (Fig 5F).

These data suggest that the occurrence of the altered breakpoint exhibited by NACC2-NTRK2(ex4:ex15), resulting in greater stability of the fusion protein, may contribute to the more severe clinical outcome of pediatric glioblastoma [10]. Removal of the transmembrane helix, either in NACC2-NTRK2-ΔTM or in NACC2-NTRK2(ex4:ex15), slows the proteasomal degradation of the fusion protein. These observations suggest that the transmembrane helix may contain a signal for degradation, known as a degron. Inspection reveals the presence of the D-box motif RxxL, characterized as a degradation signal for cyclin B1 [33]. In NTRK2, this sequence occurs as the sequence REHL at the very beginning of the TM helix, where it would normally be partly membrane embedded. The increased oncogenicity as measured by focus assays of NACC2-NTRK2(ex4:ex15) may result from deletion of this degron, leading to a longer half-life and greater ability to activate downstream signaling pathways.

## Discussion

Since the discovery of the first NTRK fusion in the 20th century, many more NTRK fusions were identified in multiple cancer types, especially in rare cancer types [32,34]. The first kinase-targeting pan-TRK inhibitor Larotrectinib was developed in 2013 and approved in 2018. Treatment with Larotrectinib yields significant results in NTRK fusion-positive cancers. To overcome the drug resistance arising from NTRK mutations, such as the NTRK3 solvent front mutation G623R [35], second-generation inhibitors are under development or in clinical trials [8] In this study, we demonstrate the importance of the BTB domain of the N-terminal fusion partner NACC2 as a potential drug target that could be utilized to overcome drug resistance in patients with NACC2-NTRK2-driven cancers.

### Characterization of NACC2-NTRK2 activation

Our experiments show that multimerization of the N-terminal BTB domain in the NACC2-NTRK2 fusion protein induces constitutive activation of the NTRK2-derived tyrosine kinase. Our downstream signaling analysis demonstrates that NACC2-NTRK2 kinase activation triggers the phosphorylation of signaling proteins in the ERK/MAPK, PLCγ1, and JAK/STAT pathways, and results in the morphological transformation of NIH3T3 cells (Fig 1B and 1D). The complete lack of activity displayed by the kinase-dead mutant, NACC2-NTRK2(K572R), demonstrates the importance of a functional NTRK2 kinase domain. Mutations which disrupt BTB-mediated multimerization, either in the charged pocket of the BTB domain or in the monomer core, exhibit diminished downstream activation, as does the complete deletion of the BTB domain (Fig 3C).

These mutants are also unable to transform NIH3T3 cells, indicating that the oncogenic activity of NACC2-NTRK2 relies not only on the constitutive activation of the tyrosine kinase domain but also requires an intact BTB domain to drive multimerization. Importantly, the diminution of P-PLCγ1 and P-STAT3 activation exhibited by the D31N/R45Q, Y86A, and ΔBTB mutants (Fig 3C and 3D) correlated with the loss of biological activity (Fig 3E).

### Deletions which remove the transmembrane helix result in increased fusion protein stability

The first case of NACC2-NTRK2(ex4:ex13) fusion was identified in pilocytic astrocytoma [9]. In 2021, a case report described a different NACC2-NTRK2 fusion with a distinct breakpoint, NACC2-NTRK2(ex4:ex15), in pediatric glioblastoma [10]. The transmembrane helix of NTRK2 emerges as a key structural motif missing from this novel fusion. Thus we constructed two different mutants to examine this more closely: NACC2-NTRK2-ΔTM which deletes the transmembrane helix, and NACC2-NTRK2(ex4:ex15) which deletes exons 13 and 14 entirely (Fig 5A). In cycloheximide chase experiments (Fig 5F), both mutants exhibit a significantly longer half-life than NACC2-NTRK2(ex4:ex13) which has a half-life of approximately 18 hours. The variant described in glioblastoma, NACC2-NTRK2(ex4:ex15), exhibited a half-life of approximately 42 hours. The NACC2-NTRK2-ΔTM variant was even more stable and did not decrease over the 20 hours of the experiment. Further experiments showed that NACC2-NTRK2-ΔTM resulted in increased downstream activation and a higher level of NIH3T3 cell transformation (Fig 5B and 5D). These data indicate that the NTRK2 transmembrane helix regulates NACC2-NTRK2 degradation, suggesting that the transmembrane helix contains a potential degron sequence [36]. By removing the transmembrane helix, the fusion protein becomes resistant to proteasomal degradation and results in more persistent kinase activation and eventually leads to higher transformation activity.

### BTB domain as a druggable target

Treatment of NTRK fusion-positive cancers currently relies on TKIs that target the NTRK tyrosine kinase activity. In this study, we demonstrate that disrupting the normal functioning of the NACC2 BTB domain could be an alternative therapeutic approach.

The BTB domain is an evolutionary conserved domain that can be found in various zinc finger proteins. The structure and function of BTB domains have been well characterized [37], but its role in NTRK fusion proteins has not been investigated. Here we demonstrate that the NACC2-NTRK2-induced signaling activity and cell transformation can be inhibited by disruption of the BTB domain of NACC2. As shown by the focus assay results of NACC2(D31N/R45Q)-NTRK2, NACC2(Y86A)-NTRK2, and NACC2(ΔBTB)-NTRK2, disruption of the BTB domain charged pocket and monomer core abrogates the cell transformation activity (Fig 3E). In our multimerization study, the NACC2(D31N/R45Q)-NTRK2 charged pocket mutation, as well as the NACC2(Y86A)-NTRK2 monomer core mutation, show reduced homo- and hetero-multimerization in addition to reduced downstream activities (Figs 2D and 3A). In another BTB domain containing fusion protein, PLZF/RAR-alpha that causes AML, similar mutations result in inactive fusion-positive cells [19]. Thus, a novel inhibitor that targets these conserved motifs may be beneficial for multiple BTB domain protein associated cancers.

Precedent of BTB domain inhibitors can be found in another BTB domain containing oncogene, B-cell lymphoma 6 (BCL6). Two types of inhibitors for this protein have been developed. BI-3802 inhibitor lead to BCL6 dimer degradation [38], and BI-3812 prevents BCL6 dimer formation [39]. Despite limitations in bioavailability, *in vitro* analysis proved their efficacy [40]. Thus, the BTB domain is a druggable domain and a novel inhibitor targeting the NACC2-NTRK2 BTB domain charged pocket or monomer core can be envisioned as an alternative treatment method to overcome possible TKI resistance.

The study of a shorter form of the fusion protein BTB-NTRK2 reveals another interesting aspect of NACC2 in the fusion protein. In the homo-multimerization experiment, protein samples were prepared in non-reducing sample buffer and resolved in SDS-PAGE. The NACC2-NTRK2 fusion and BTB domain point mutants were still able to multimerize whereas the homo-multimerization of BTB-NTRK2 was not observed (Fig 4D). However, the coimmunoprecipitation experiment proved that BTB-NTRK2 was able to interact with the BTB domain of native NACC2 as does NACC2-NTRK2 (Fig 4E, 3^rd^ panel). Also, BTB-NTRK2 focus assay results showed that the BTB domain alone, in BTB-NTRK2, is able to mediate downstream activation and transform NIH3T3 cells, although at a reduced level (Fig 4F). These data together indicate that NACC2 residues in the disordered region (residues 121–350), possibly together with the residual BEN domain (residues 351–418), provide structural support for NACC2-mediated multimerization through covalent interactions, making it resistant to SDS denaturation. Since this multimer is sensitive to β-mercaptoethanol reduction (Fig 2D), this establishes the existence of one or more intermolecular disulfide bonds stabilizing the multimer. This retained portion of NACC2 from residues 351–418 includes a total of seven Cys residues: four in the disordered region, and three in the partial BEN domain. It will be interesting in future experiments to determine which of these seven residues are responsible for this covalent disulfide bonding. In summary, the BTB domain itself is responsible for self-association–a precondition for multimerization–but other sequences, contributed by NACC2 and which lie downstream of the BTB domain, provide a major enhancement to structural stability and biological activity.

These data also suggest, for NTRK2 fusion proteins, that domains other than the multimerization domain in the N-terminal fusion partner may contribute important regulatory functions. For example, in the QKI-NTRK2 fusion there exist three domains from QKI: the Qua1 domain responsible for protein dimerization, the KH domain that mediates RNA recognition, and the Qua2 domain that interacts with RNA [41]. As a homodimerization domain, the Qua1 domain fulfils a similar function in NTRK2 activation as the BTB domain; however, the other two domains, KH and Qua2, may contribute additional regulatory functions to the QKI-NTRK2 fusion. Thus, it will be interesting to investigate the roles of protein domains within the N-terminal fusion partners above and beyond multimerization.

Through this study, we briefly characterized the functions of every domain in the NACC2-NTRK2. The BTB domain (1–120) is responsible for fusion protein multimerization and the residues 121–418 are responsible for stabilization of the multimer. In the NTRK2-derived portion, the kinase domain is responsible for the oncogenic activation of the fusion, and lastly, removal of the transmembrane helix results in greater stability of the fusion protein and higher-grade activation. Point mutations in the NACC2 BTB domain abrogate the activity of the NACC2-NTRK2 fusion protein. Thus, patients may benefit from a combined treatment of TKI inhibition and BTB domain inhibition. These findings suggest that characterizations of oncogenic fusion proteins are important for the discovery of novel therapeutic methods and facilitate the development of novel treatments in the battle against fusion-positive cancers.

## Materials and methods

### DNA constructs

Genes for NACC2 (SC319661) and NTRK2 (RG221838) were purchased from Origene (Rockville, MD, USA). NTRK2 1770G was found to be missing in RG221838; this was repaired by site-directed mutagenesis. To subclone both genes into pCDNA3 vector, an XbaI restriction site was introduced at the 3’ ends of both NACC2 and NTRK2 by quick-change PCR mutagenesis. NACC2 and NTRK2 were subcloned into pCDNA3 vector using upstream EcoRI sites and the introduced XbaI sites. A ClaI restriction site was introduced at residue V417 of NACC2 and Y397 of NTRK2. NACC2 and NTRK2 were joined together following ClaI and XbaI double digestion and ligation. Point mutations (D31N/R45Q, Y86A, K572R) were introduced by site-directed PCR mutagenesis. Domain deletions (ΔBTB, Δ121–418, ΔTM, and (ex4:ex15)) were introduced by PCR site-directed mutagenesis [42]. For NIH3T3 transformation assays, DNA constructs were subcloned into the murine retroviral vector pLXSN [43] using EcoRI and XbaI restriction sites. All NACC2-NTRK2 genes expressed in pcDNA3 clones are identical to those expressed in pLXSN clones, and all maintain an identical sequence surrounding the initiation codon ATG. The only difference is that in pcDNA3 vectors, genes are expressed from a Human Cytomegalovirus (CMV) immediate-early promoter for high-level expression [44], whereas in pLXSN vectors, genes are expressed from the Moloney Murine Leukemia Virus (MoMLV) LTR promoter for stable long-term expression [43].

### Antibodies and reagents

Antibodies were purchased as following: NACC2 (Bethyl A304-991A), P-NTRK (Cell Signaling 4621S), NTRK2 (Invitrogen PA5-86241), P-MAPK (Cell Signaling 4370), MAPK (Cell Signaling 9102), P-PLCγ1 (Cell Signaling 2821), PLCγ1 (Santa Cruz sc-81), P-STAT3 (Cell Signaling 9145), STAT3 (Cell Signaling 9139). Enhanced Chemiluminescence (ECL) reagents, horseradish peroxidase (HRP) anti-mouse (NA931V) and HRP anti-rabbit (NA934V) secondary antibodies were purchased from Cytiva.

Other reagents were purchased as following: G-418 Sulfate (Fisher Scientific), Lipofectamine 2000 (Invitrogen), Protein A-Sepharose (Sigma P3391), and Cycloheximide (Fisher J66901.03).

### Transfection and immunoblotting

HEK293T cells were grown in 1% Pen/Strep and 10% FBS and seeded at 10^6^ cells per 10 cm plate. 5 μg of pCDNA3 DNA constructs were transfected using CaCl_2_ transfection as described previously [25]. Cells were incubated in 3% CO2 for 14–18 hours, recovered in 10% CO2 for 6–8 hours, and then starved with 0% FBS media for 14–16 hours. Cells were lysed in RIPA buffer [50 mM Tris HCl pH 8.0, 150 mM NaCl, 1% Triton X-100, 0.5% sodium deoxycholate, 0.1% SDS, 50 mM NaF] with 1 mM sodium orthovanadate, 1 mM PMSF and 10 μg/mL aprotinin. Lysate concentrations were determined by Lowry Assay. Samples were resolved by SDS-PAGE and transferred to Immobilon-P PVDF membranes (Millipore, Burlington, MA, USA). Membranes were blocked with either 5% BSA in 0.1% TBS-T or 5% nonfat milk in 0.1%TBS-T.

### Immunoprecipitation and multimerization assay

DNA constructs were transfected into HEK293T cells using CaCl_2_ transfection as above and collected with E1A buffer [250 mM NaCl, 50 mM HEPES, 5 mM EDTA and 0.1% NP-40] [30]. Total protein concentrations were measured by Lowry Assay. For multimerization assay, protein samples were prepared with either reducing sample buffer (50mM Tris-Cl, 10% β-mercaptoethanol, 2% SDS, 0.1% bromophenol blue, 10% glycerol) or non-reducing sample buffer (4% SDS,10 mM NaPO4 pH7.0, 20% glycerol, 0.08% Bromophenol blue). Samples were resolved by SDS-PAGE. For immunoprecipitation, 300 μg of total protein was diluted to 1mL with E1A wash buffer [125 mM NaCl, 50 mM HEPES, 0.2% NP-40 and 5 mM EDTA]. Diluted lysates were precleared with Protein A-Sepharose for 3 hours rocking at 4°C, and then incubated with primary antibody overnight at 4°C. Protein A-Sepharose was added to recover immune complexes. Beads were washed with E1A wash buffer 5 times followed by addition of 30 μL of reducing sample buffer. Samples were resolved by SDS-PAGE and immunoblotted as above.

### Focus assay

NIH3T3 cells were maintained in DMEM with 10% calf serum and 1% penicillin/streptomycin in 10% CO_2_ at 37 degC Focus assays were performed basically as described [45]. NIH3T3 cells were plated at a density of 4 x 10^5^ cells on 60mm plates 24 hours before transfection. Cells were transfected by Lipofectamine 2000 Reagent with 10 μg of pLXSN DNA constructs. Approximately 24 hours after transfection, cells were re-fed with DMEM + 10% calf serum. After an additional 24 hours, cells were split 1:12 onto duplicate 100-mm plates later containing 2.5% calf serum for the focus forming assays, and 1:240 onto duplicate 100-mm plates containing 10% calf serum and Geneticin (G418, 0.5 mg/mL), in order to measure the expression and efficiency of the transfections. After 14 days, focus assay plates were fixed in methanol, stained with Geimsa stain, and scored. Geneticin-resistant colonies were also fixed with methanol after 14 days and scored. Numbers of foci were normalized to the number of G418-resistant colonies, and quantitated relative to a positive control–/+ standard error of the mean (SEM).

### Cycloheximide chase

HEK293T cells were seeded at 10^6^ cells per 10 cm plate and transfected with 2–5 μg of pcDNA3 DNA constructs. After recovery at 10% CO_2_ for 6–8 hours, cell were treated with 300 μg/ml of cycloheximide in ethanol for 0, 5,10 and 20 hours. Cells were harvested and lysed in RIPA.

## Supporting information

S1 Raw images (PDF)

S1 Data (XLSX)
