# Supplementary material for: Critical domains for NACC2-NTRK2 fusion protein activation
Source: PLoS One. 2024 Jun 27;19(6):e0301730. doi: 10.1371/journal.pone.0301730 (PMC11210774; doi:10.1371/journal.pone.0301730)
Supplement: S1 Raw images — (PDF) [file pone.0301730.s001.pdf]

Critical Domains for NACC2-NTRK2 Fusion Protein Activation

Authors: Wei Yang, April N. Meyer, Zian Jiang, Xuan Jiang, and Daniel J. Donoghue

Article in review for publication in PLoS One

Native Immunoblots for Figure 1D

Legend to Figure 1. NACC2-NTRK2 exhibits biological activity in cell transformation and cell signaling assays.

(D) The activation of downstream signaling pathways is presented. HEK293T cell lysates expressing NTRK2, NTRK2(KD), NACC2-NTRK2, and NACC2-NTRK2(KD) were analyzed by SDS-PAGE and immunoblotted for P-NTRK (top panel), total NTRK2 (2nd panel), P-MAPK (3nd panel), total MAPK (4th panel), P-PLCγ1 (5th panel, upper band), total PLCγ1(6th panel), P-STAT3(7th panel) and total STAT3(8th panel). Note that in this figure, and other immunoblots of P-PLCγ1, the band of interest migrates above the 130 kD marker; the prominent band near the bottom of this blot represents antiserum cross-reactivity with the activated NTRK2 kinase domain.

Native Immunoblots for Figure 1D are presented on the following pages

Figure 1D
Panel 1
P-NTRK2

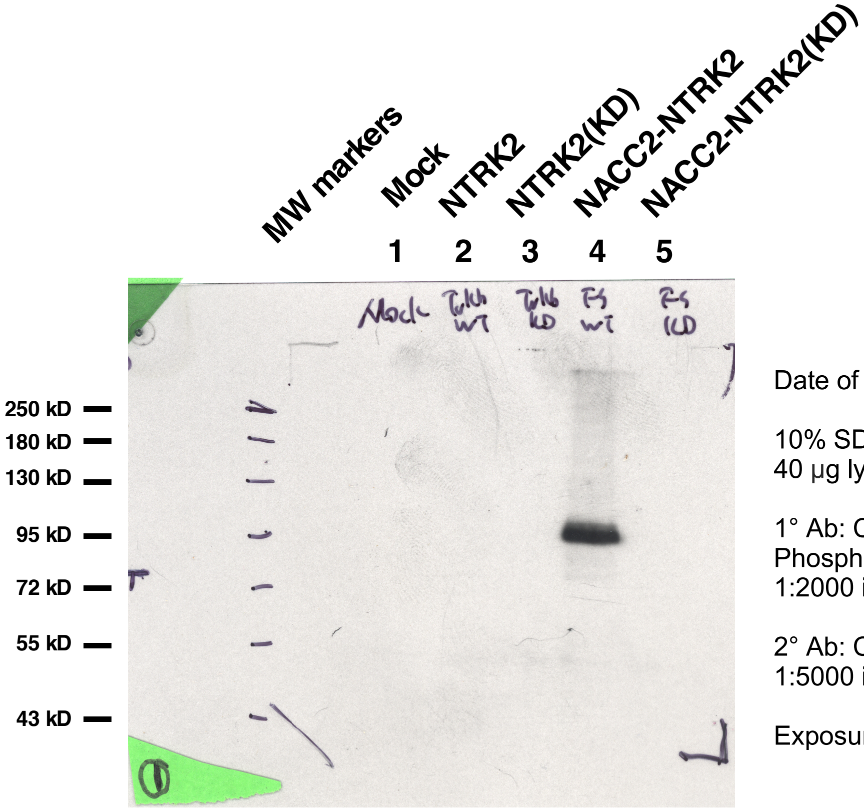

Date of immunoblot: 9/7/2022

10% SDS-PAGE, 180 V
40 µg lysate per lane

1° Ab: Cell Signaling #4621
Phospho-TrkA/TrkB Rabbit mAb
1:2000 in 5% BSA, 0.1% TBS-T

2° Ab: Cytiva NA934V HRP αRabbit
1:5000 in 5% BSA, 0.1% TBS-T

Exposure time: 2 min

Figure 1D
Panel 2
NTRK2

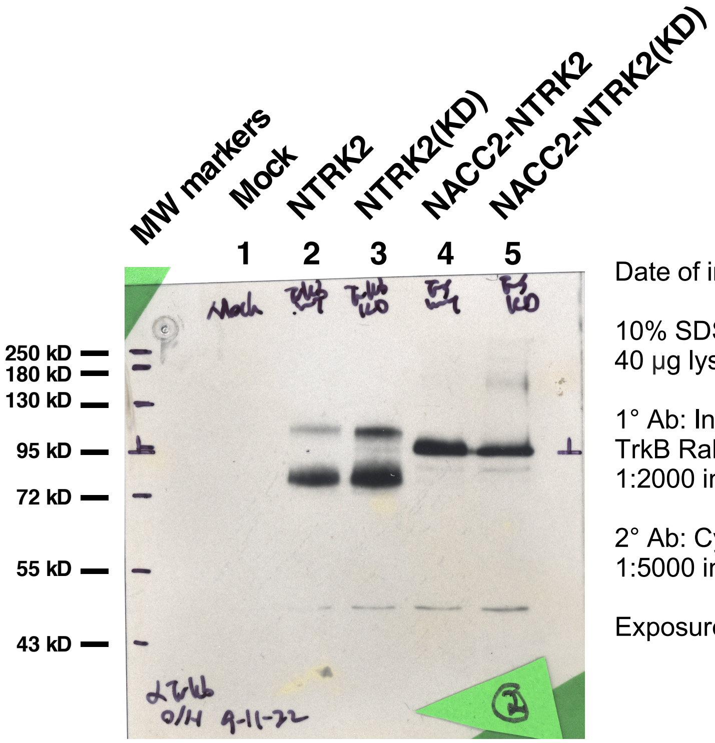

Date of immunoblot: 9/11/2022

10% SDS-PAGE, 180 V
40 µg lysate per lane

1° Ab: Invitrogen PA5-86241
TrkB Rabbit polyclonal Ab
1:2000 in 5% BSA, 0.1% TBS-T

2° Ab: Cytiva NA934V HRP αRabbit
1:5000 in 5% milk, 0.1% TBS-T

Exposure time: Overnight

Figure 1D
Panel 3
P-MAPK

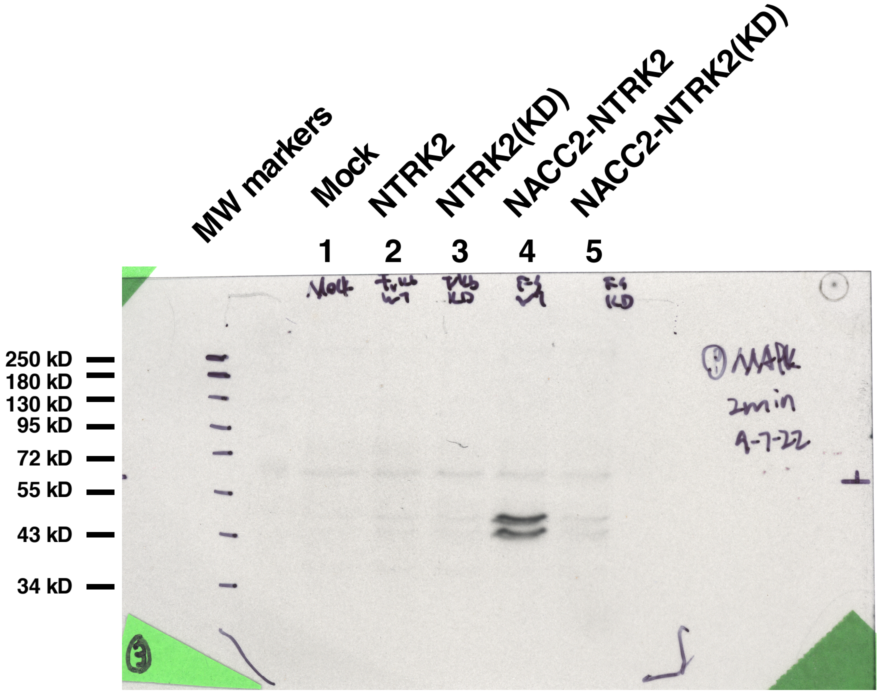

Date of immunoblot: 9/7/2022

12.5% SDS-PAGE, 180 V
40 µg lysate per lane

1° Ab: Cell Signaling #4370
Phospho-p44/42 MAPK (Erk1/2)
Rabbit mAb (Thr202/Tyr204)
(D13.14.4E)
1:2000 in 5% BSA, 0.1% TBS-T

2°Ab: Cytiva NA934V HRP αRabbit
1:2000 in 5% BSA, 0.1% TBS-T

Exposure time: 2 min

Figure 1D
Panel 4
MAPK

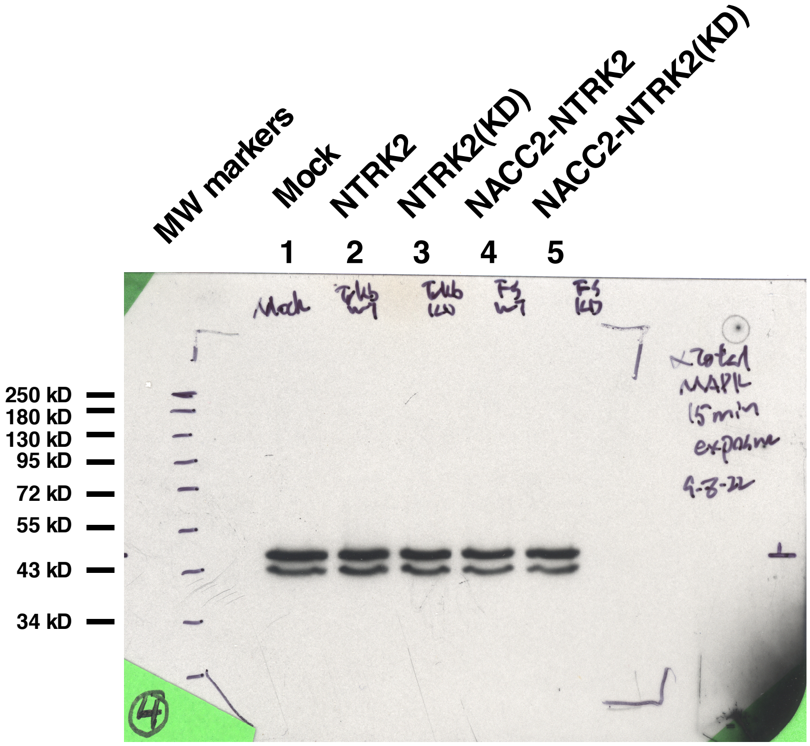

Date of immunoblot: 9/8/2022

12.5% SDS-PAGE, 180 V
40 µg lysate per lane

1° Ab: Cell Signaling #9102
p44/42 MAPK (Erk1/2)
Rabbit polyclonal Ab
1:2000 in 5% BSA, 0.1% TBS-T

2°Ab: Cytiva NA934V HRP αRabbit
1:2000 in 5% BSA, 0.1% TBS-T

Exposure time: 15 min

Figure 1D
Panel 5
P-PLCγ1

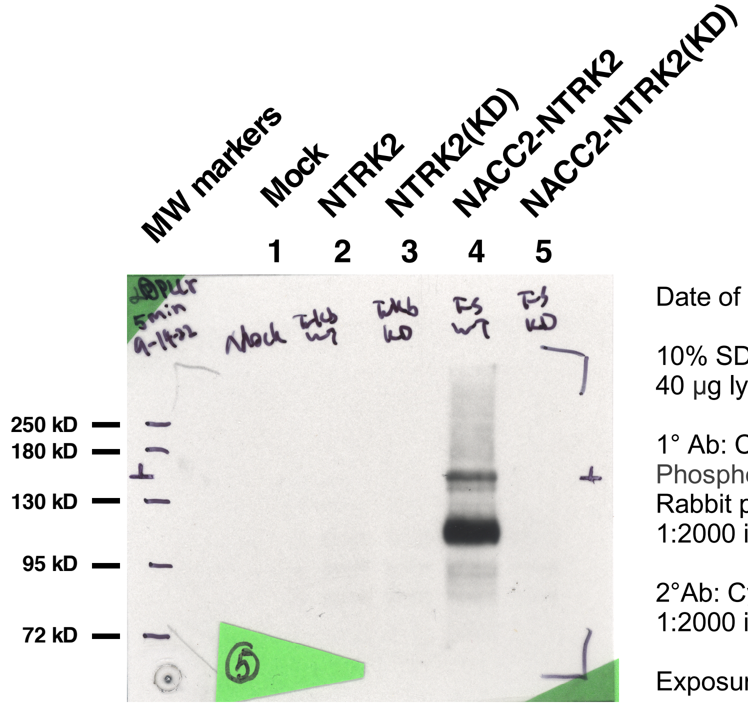

Date of immunoblot: 9/14/2022

10% SDS-PAGE, 180 V
40 µg lysate per lane

1° Ab: Cell Signaling #2821
Phospho-PLCγ1 (Tyr783) Antibody
Rabbit polyclonal AB
1:2000 in 5% BSA, 0.1% TBS-T

2°Ab: Cytiva NA934V HRP αRabbit
1:2000 in 5% BSA, 0.1% TBS-T

Exposure time: 5 min

Figure 1D
Panel 6
PLCγ1

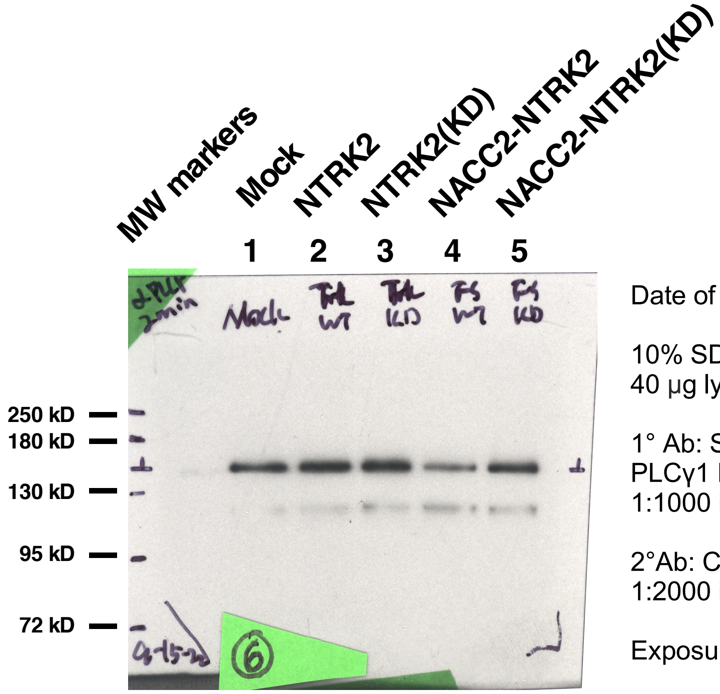

Date of immunoblot: 9/15/2022

10% SDS-PAGE, 180 V
40 µg lysate per lane

1° Ab: Santa Cruz (1249) sc-81
PLCγ1 Rabbit polyclonal Ab
1:1000 in 5% BSA, 0.1% TBS-T

2°Ab: Cytiva NA934V HRP αRabbit
1:2000 in 5% BSA, 0.1% TBS-T

Exposure time: 2 min

Figure 1D
Panel 7
P-STAT3

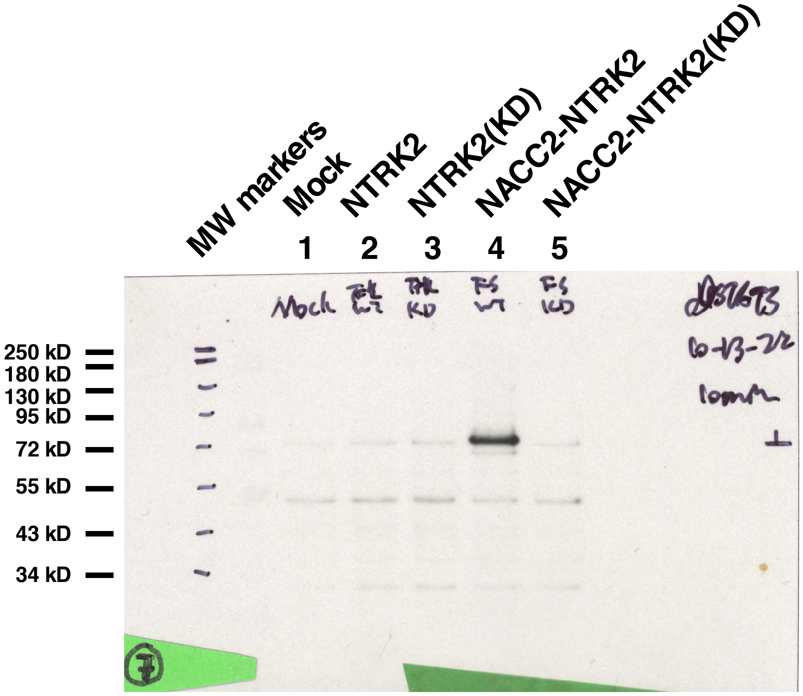

Date of immunoblot: 10/13/2022

10% SDS-PAGE, 180 V
40 μg lysate per lane

1° Ab: Cell Signaling #9145
Phospho-Stat3 (Tyr705) (D3A7)
Rabbit mAb
1:1000 in 5% BSA, 0.1% TBS-T

2°Ab: Cytiva NA934V HRP αRabbit
1:2000 in 5% BSA, 0.1% TBS-T

Exposure time: 10 min

Figure 1D
Panel 8
STAT3

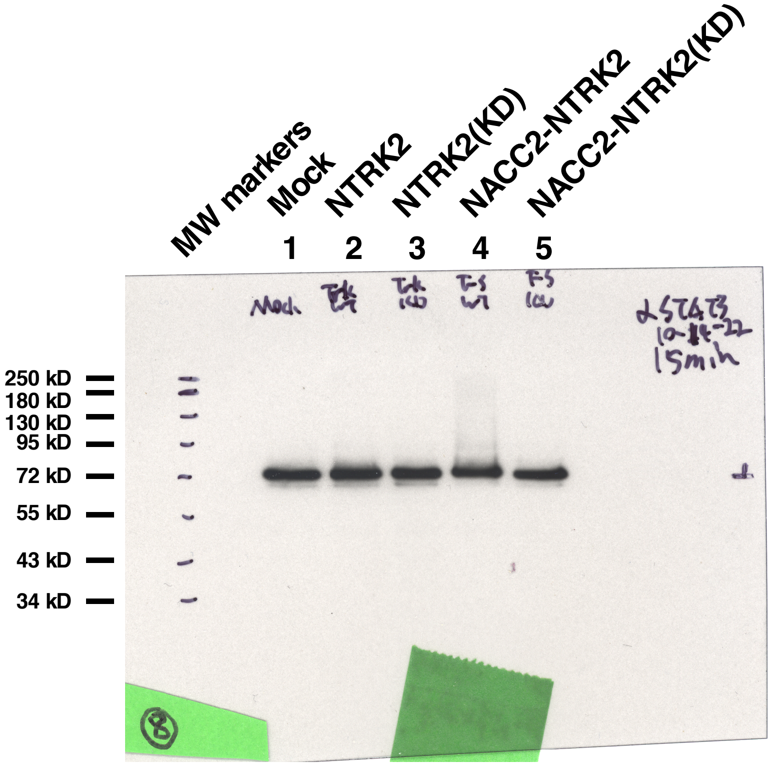

Date of immunoblot: 10/14/2022

10% SDS-PAGE, 180 V
40 μg lysate per lane

1° Ab: Cell Signaling #9139
Stat3 (124H6) Mouse mAb
1:500 in 5% BSA, 0.1% TBS-T

2°Ab: Cytiva NA931V HRP αMouse
1:2000 in 5% BSA, 0.1% TBS-T

Exposure time: 15 min

Critical Domains for NACC2-NTRK2 Fusion Protein Activation
Authors: Wei Yang, April N. Meyer, Zian Jiang, Xuan Jiang, and Daniel J. Donoghue
Article in review for publication in PLoS One

Native Immunoblots for Figure 2D

Legend to Figure 2. The BTB domain of NACC2 is responsible for multimerization of NACC2-NTRK2.

(D) Lysates from HEK293T cells expressing NACC2, NACC2-NTRK2, NACC2(D31N/R45Q)-NTRK2, and NACC2(Y86A)-NTRK2 were analyzed by SDS-PAGE and immunoblotted for NACC2. Non-reducing sample buffer (left panel) or reducing sample buffer (right panel) was used for duplicate samples. Significant multimer bands of NACC2 and NACC2-NTRK2 were observed in the non-reducing conditions (left panel, lanes 2 and 3).

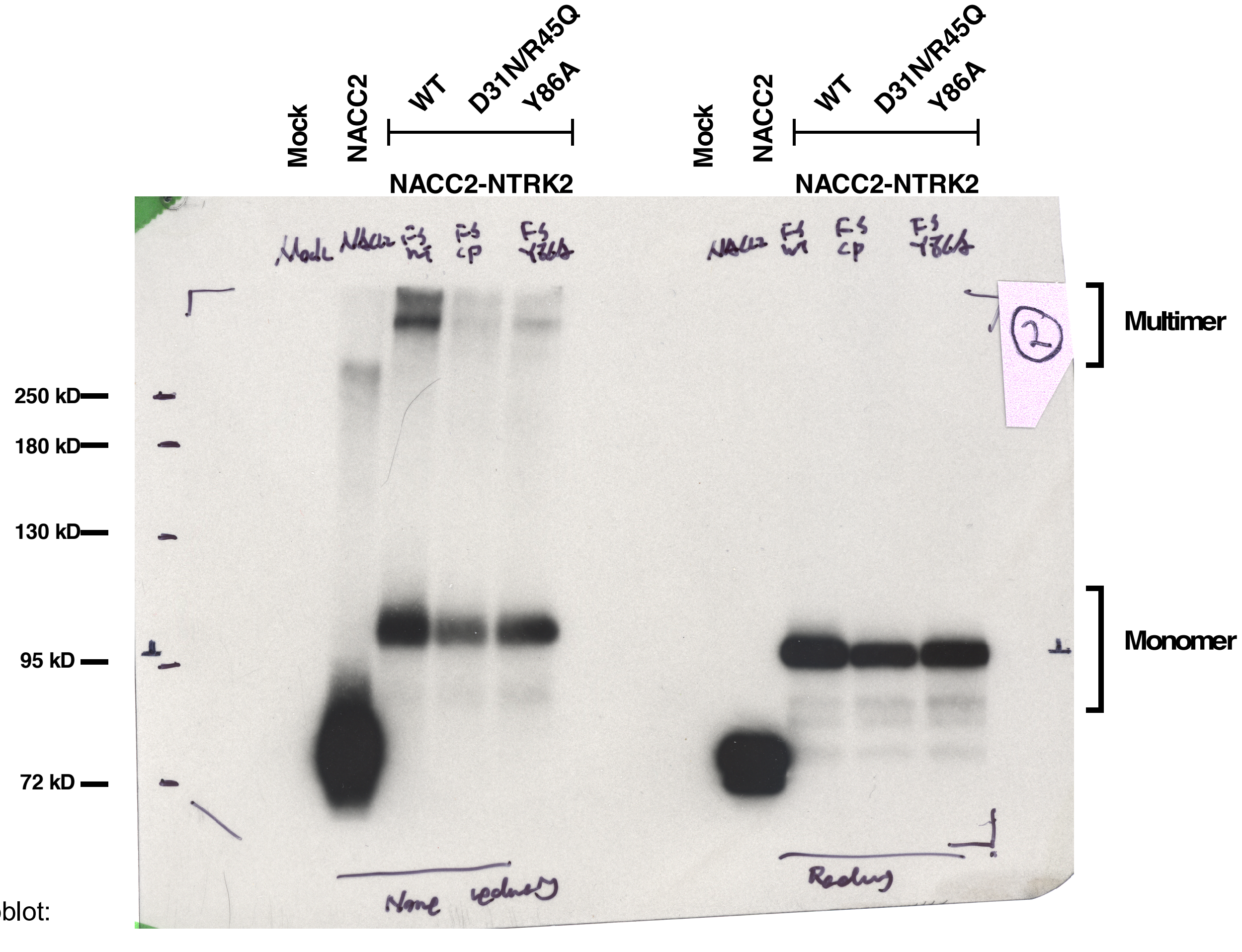

Date of immunoblot:
2/2/2023

7.5% SDS-PAGE large gel
50V O/N

1° Ab: Bethyl A304-991A
Rabbit anti-NACC2/BTBD14A
Polyclonal Ab
1:2000 in 5% BSA, 0.1% TBS-T

2° Ab: Cytiva NA934V
HRP αRabbit
1:5000 in 0.5% milk, 0.1% TBS-T

Exposure time: 1 min

Multimer Assay

Figure 2D
Left Panel
IB: NACC2
Non-reducing
Samples loaded in:
4% SDS,
10 mM NaPO4 pH7.0,
0.08% Bromophenol blue,
20% glycerol

Reducing SDS-PAGE

Figure 2D
Right Panel
IB: NACC2
Reducing
Samples loaded in:
2% SDS,
50mM Tris-Cl,
10% β-mercaptoethanol,
0.1% bromophenol blue,
10% glycerol

Critical Domains for NACC2-NTRK2 Fusion Protein Activation

Authors: Wei Yang, April N. Meyer, Zian Jiang, Xuan Jiang, and Daniel J. Donoghue

Native Immunoblots for Figure 3A

Legend to Figure 3. The BTB domain of NACC2 is required for biological activity of NACC2-NTRK2.

(A) The interaction of NACC2 and NACC2-NTRK2 is examined. Lysates of HEK293T cells expressing NACC2-NTRK2 (designated as WT), or with the mutations D31N/R45Q or Y86A, were coexpressed with NACC2 as indicated (Lanes 4-6). Samples were prepared in E1A buffer to preserve protein-protein interactions. The 3rd panel presents the results of immunoprecipitation using antibodies against NTRK2, to recover the NACC2-NTRK fusion proteins, followed by immunoblotting to detect NACC2 present in the immune complexes. Lane 4 clearly shows binding of NACC2 to NACC2-NTRK2, which is reduced in the presence of the mutations D31N/R45Q (lane 5), or Y86A (lane 6). Control immunoblots are shown for NTRK2 (top panel) and NACC2 (middle panel).

Native Immunoblots for Figure 3A are presented on the following page

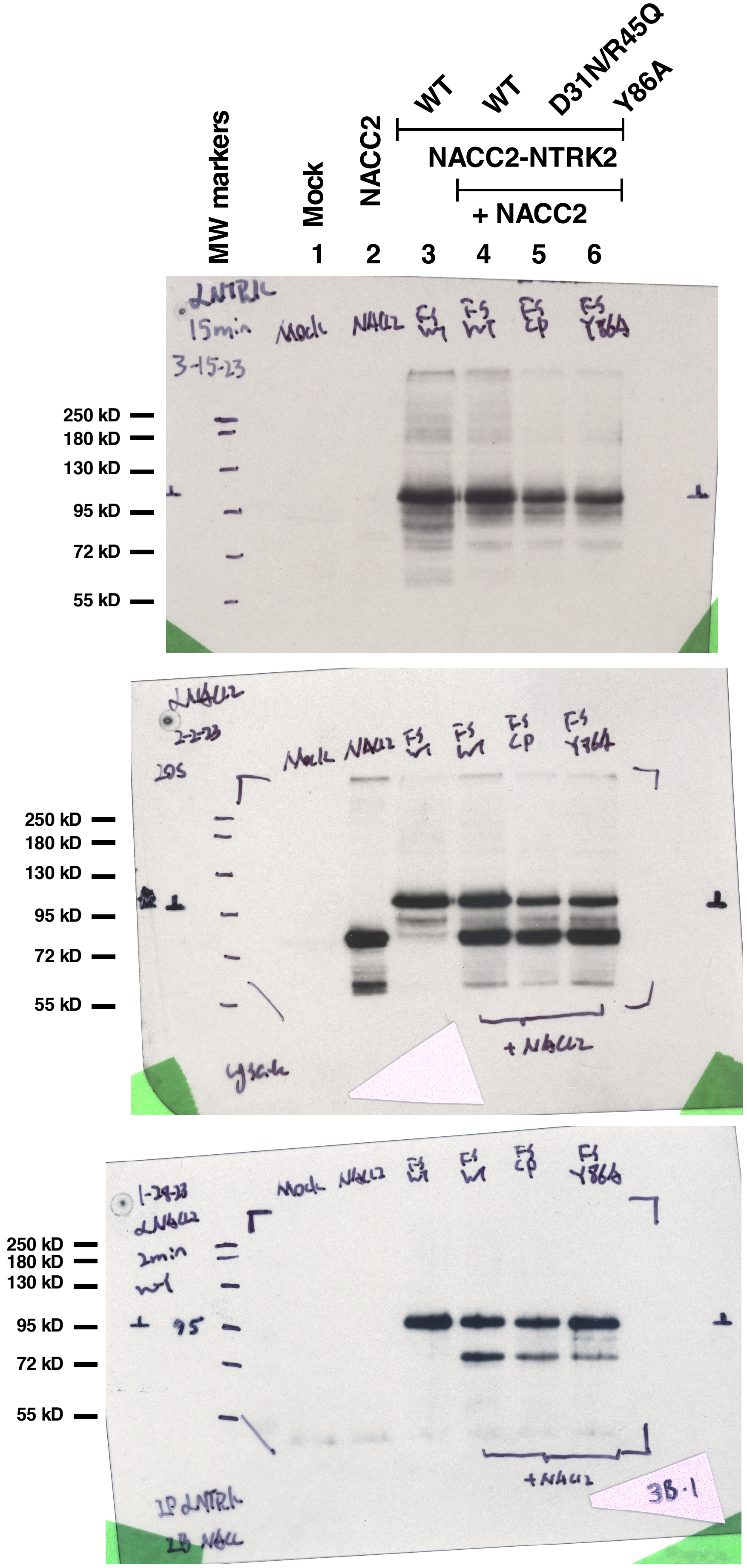

Figure 3A
Top Panel
IB: NTRK2

Date of immunoblot: 3/15/2023

10% SDS-PAGE, 180 V
40 µg lysate per lane

1° Ab: Invitrogen #PA5-86241
TrkB Rabbit Polyclonal Ab
1:2000 in 5% BSA, 0.1% TBS-T

2° Ab: Cytiva NA934V HRP αRabbit
1:5000 in 5% milk, 0.1% TBS-T

Exposure time: 15 min

Figure 3A
Middle Panel
IB: NACC2

Date of immunoblot: 2/2/2023

10% SDS-PAGE, 180 V
40 µg lysate per lane

1° Ab: Bethyl A304-991A
Rabbit anti-NACC2/BTBD14A
Polyclonal Ab
1:2000 in 5% BSA, 0.1% TBS-T

2° Ab: Cytiva NA934V HRP αRabbit
1:5000 in 0.5% milk, 0.1% TBS-T

Exposure time: 20 sec

Figure 3A
Bottom Panel
IP: NTRK2
IB: NACC2

Date of immunoblot: 1/28/2023

10% SDS-PAGE, 180 V

IP Ab: Invitrogen #PA5-86241
TrkB Rabbit Polyclonal Ab
300µg total protein per sample IP'd
with 600ng of anti-TrkB antibody

1° Ab: Bethyl A304-991A
Rabbit anti-NACC2/BTBD14A
Polyclonal Ab
1:2000 in 5% BSA, 0.1% TBS-T

2° Ab: Cytiva NA934V HRP αRabbit
1:5000 in 0.5% milk, 0.1% TBS-T

Exposure time: 2 min

Critical Domains for NACC2-NTRK2 Fusion Protein Activation

Authors: Wei Yang, April N. Meyer, Zian Jiang, Xuan Jiang, and Daniel J. Donoghue

Article in review for publication in PLoS One

Native Immunoblots for Figure 3C

Figure 3. The BTB domain of NACC2 is required for biological activity of NACC2-NTRK2.

(C) HEK293T cells expressing NACC2-NTRK2, NACC2-NTRK2(KD), NACC2(D31N/R45Q)-NTRK2, NACC2(Y86A)-NTRK2 or NACC2(ΔBTB)-NTRK2 were examined for the activation of downstream signaling pathways. Cell lysates were analyzed by SDS-PAGE and immunoblotted for P-NTRK (top panel), total NTRK2 (2nd panel), P-MAPK (3nd panel), total MAPK (4th panel), P-PLCγ1 (5th panel, upper band), total PLCγ1 (6th panel), P-STAT3(7th panel) and total STAT3(8th panel).

Native Immunoblots for Figure 3C are presented on the following pages

Figure 3C
Panel 1
P-NTRK2

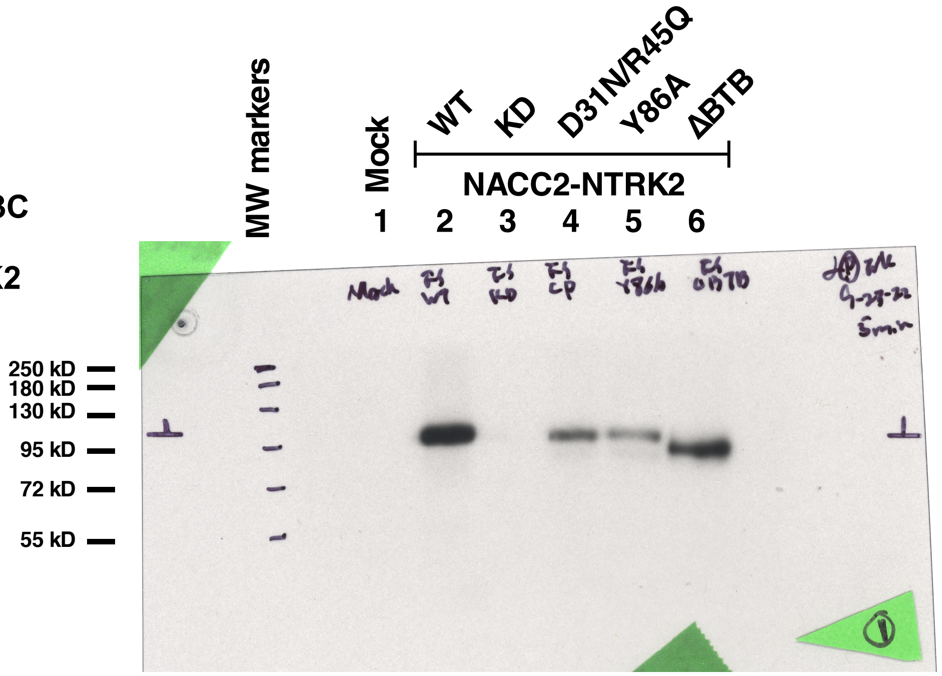

Date of immunoblot: 9/23/2022

10% SDS-PAGE, 180 V
40 µg lysate per lane

1° Ab: Cell Signaling #4621
Phospho-TrkA/TrkB Rabbit mAb
1:2000 in 5% BSA, 0.1% TBS-T

2° Ab: Cytiva NA934V HRP αRabbit
1:5000 in 5% BSA, 0.1% TBS-T

Exposure time: 5 min

Figure 3C
Panel 2
NTRK2

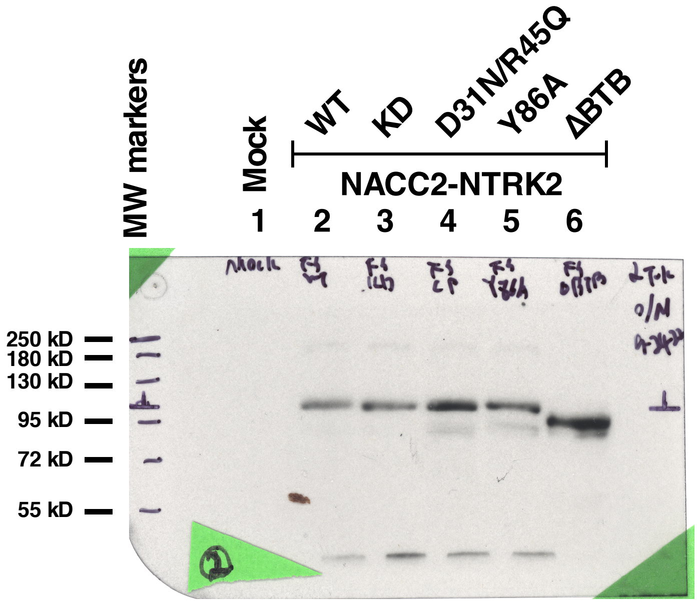

Date of immunoblot: 9/24/2022

10% SDS-PAGE, 180 V
40 µg lysate per lane

1° Ab: Invitrogen PA5-86241
TrkB Rabbit polyclonal Ab
1:2000 in 5% BSA, 0.1% TBS-T

2° Ab: Cytiva NA934V HRP αRabbit
1:5000 in 5% milk, 0.1% TBS-T

Exposure time: Overnight

Figure 3C
Panel 3
P-MAPK

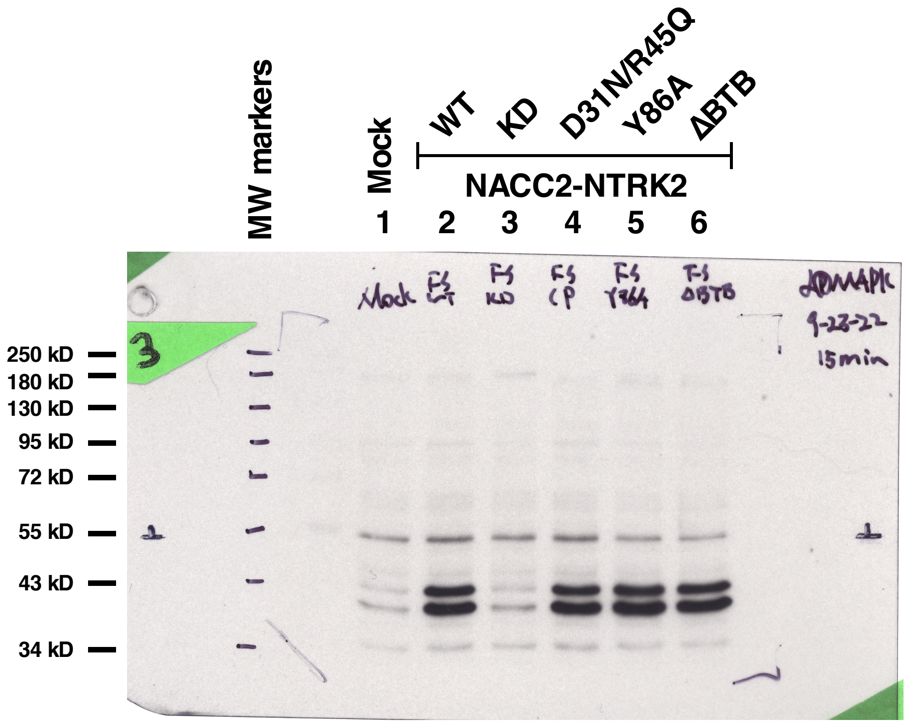

Date of immunoblot: 9/23/2022

12.5% SDS-PAGE, 180 V
40 µg lysate per lane

1° Ab: Cell Signaling #4370
Phospho-p44/42 MAPK (Erk1/2)
Rabbit mAb (Thr202/Tyr204)
(D13.14.4E)
1:2000 in 5% BSA, 0.1% TBS-T

2°Ab: Cytiva NA934V HRP αRabbit
1:2000 in 5% BSA, 0.1% TBS-T

Exposure time: 15 min

Figure 3C
Panel 4
MAPK

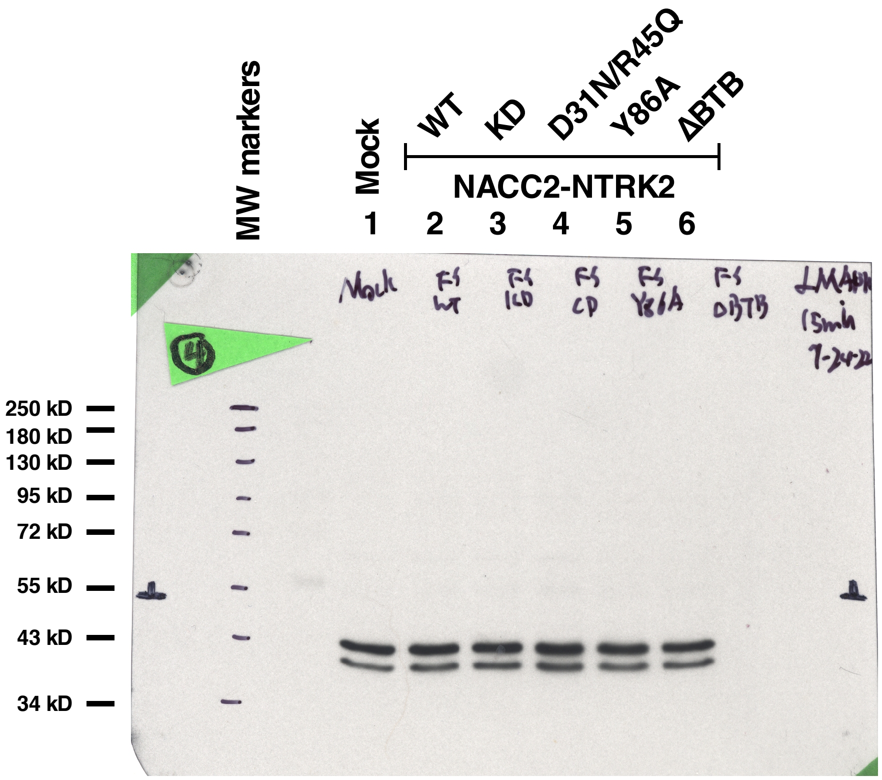

Date of immunoblot: 9/24/2022

12.5% SDS-PAGE, 180 V
40 µg lysate per lane

1° Ab: Cell Signaling #9102
p44/42 MAPK (Erk1/2)
Rabbit polyclonal Ab
1:2000 in 5% BSA, 0.1% TBS-T

2°Ab: Cytiva NA934V HRP αRabbit
1:2000 in 5% BSA, 0.1% TBS-T

Exposure time: 15 min

Figure 3C
Panel 5
P-PLCγ1

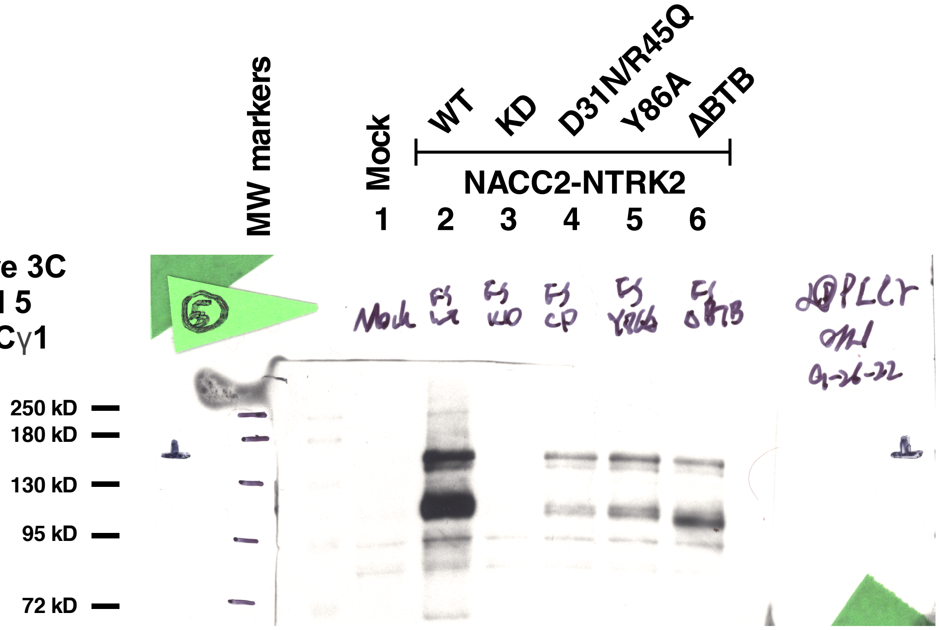

Date of immunoblot: 9/26/2022

10% SDS-PAGE, 180 V
40 µg lysate per lane

1° Ab: Cell Signaling #2821
Phospho-PLCγ1 (Tyr783) Antibody
Rabbit polyclonal AB
1:2000 in 5% BSA, 0.1% TBS-T

2°Ab: Cytiva NA934V HRP αRabbit
1:2000 in 5% BSA, 0.1% TBS-T

Exposure time: O/N

Figure 3C
Panel 6
PLCγ1

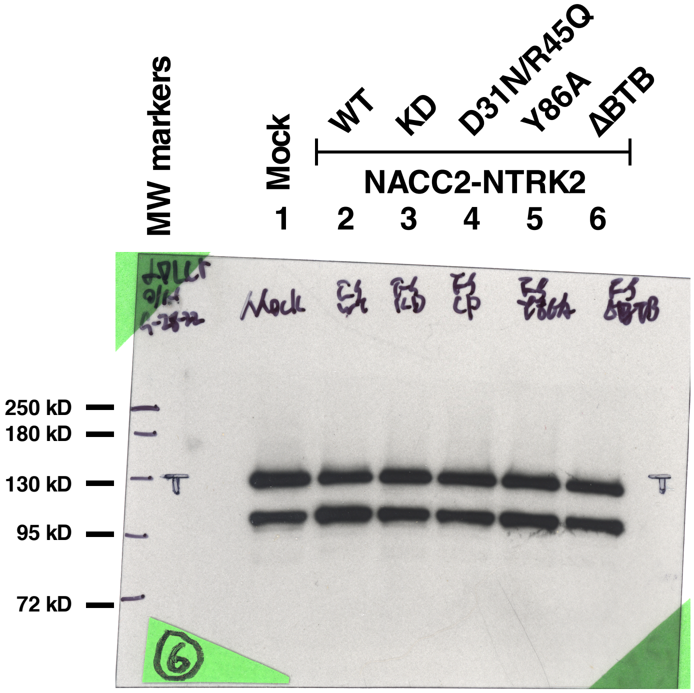

Date of immunoblot: 9/28/2022

10% SDS-PAGE, 180 V
40 µg lysate per lane

1° Ab: Santa Cruz (1249) sc-81
PLCγ1 Rabbit polyclonal Ab
1:1000 in 5% BSA, 0.1% TBS-T

2°Ab: Cytiva NA934V HRP αRabbit
1:2000 in 5% BSA, 0.1% TBS-T

Exposure time: O/N

Figure 3C
Panel 7
P-STAT3

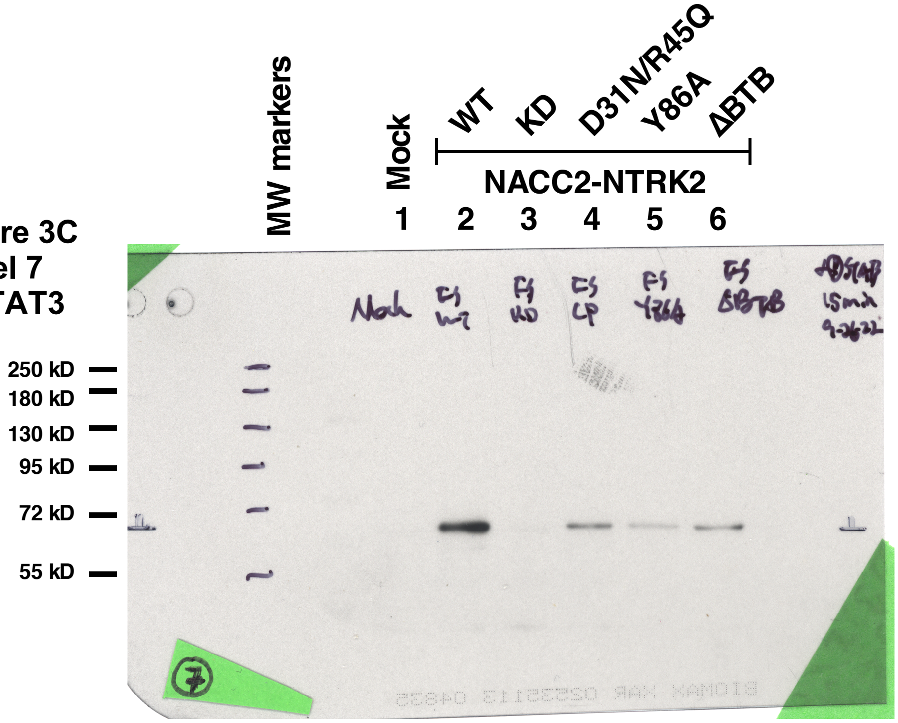

Date of immunoblot: 9/26/2022

10% SDS-PAGE, 180 V
40 µg lysate per lane

1° Ab: Cell Signaling #9145
Phospho-Stat3 (Tyr705) (D3A7)
Rabbit mAb
1:1000 in 5% BSA, 0.1% TBS-T

2°Ab: Cytiva NA934V HRP αRabbit
1:2000 in 5% BSA, 0.1% TBS-T

Exposure time: 15 min

Figure 3C
Panel 8
STAT3

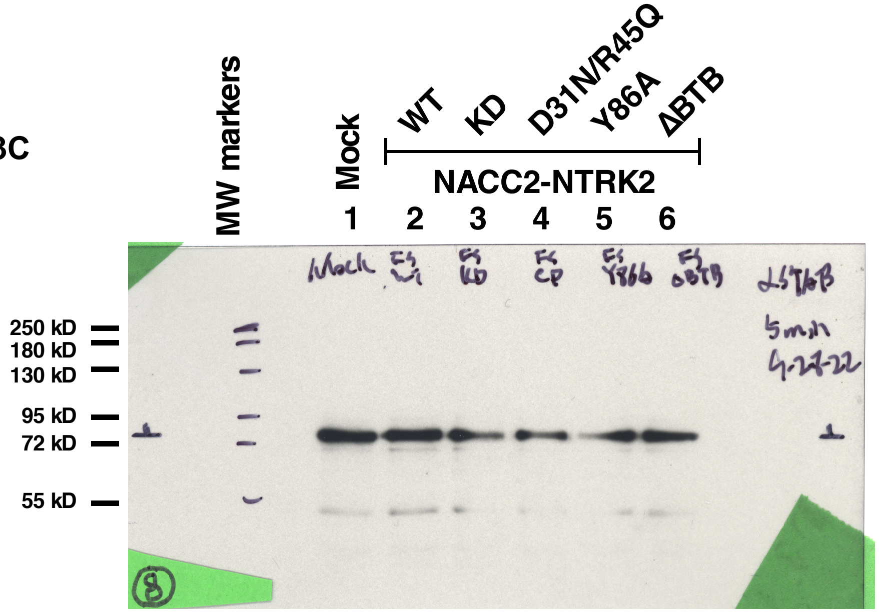

Date of immunoblot: 9/27/2022

10% SDS-PAGE, 180 V
40 µg lysate per lane

1° Ab: Cell Signaling #9139
Stat3 (124H6) Mouse mAb
1:500 in 5% BSA, 0.1% TBS-T

2°Ab: Cytiva NA931V HRP αMouse
1:2000 in 5% BSA, 0.1% TBS-T

Exposure time: 5 min

Critical Domains for NACC2-NTRK2 Fusion Protein Activation

Authors: Wei Yang, April N. Meyer, Zian Jiang, Xuan Jiang, and Daniel J. Donoghue

Article in review for publication in PLoS One

Native Immunoblots for Figure 4B

Legend to Figure 4. NACC2 residues 121-418 provides potential covalent stabilization for NACC2-NTRK2 multimer.

(B) Downstream activation by BTB-NTRK2. HEK293T cells expressing NACC2-NTRK2 or BTB-NTRK2 were examined for activation of downstream signaling pathways, as described previously for Figures 1D and 3C.

Native Immunoblots for Figure 4B are presented on the following pages

Figure 4B
Panel 1
P-NTRK2

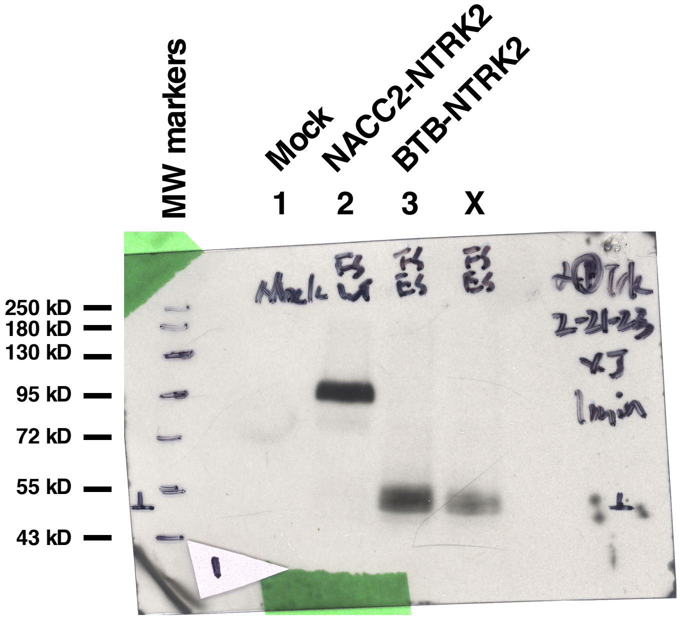

Date of immunoblot: 2/21/2023

10% SDS-PAGE, 180 V
40 μg lysate per lane

1° Ab: Cell Signaling #4621
Phospho-TrkA/TrkB Rabbit mAb
1:2000 in 5% BSA, 0.1% TBS-T

2° Ab: Cytiva NA934V HRP αRabbit
1:5000 in 5% BSA, 0.1% TBS-T

Exposure time: 1 min

Figure 4B
Panel 2
NTRK2

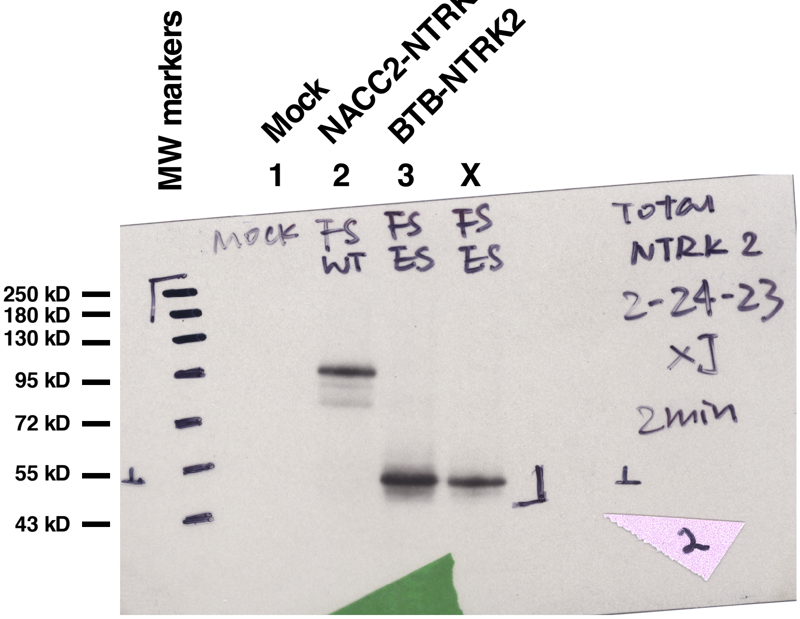

Date of immunoblot: 2/24/2023

10% SDS-PAGE, 180 V
40 μg lysate per lane

1° Ab: Invitrogen PA5-86241
TrkB Rabbit polyclonal Ab
1:2000 in 5% BSA, 0.1% TBS-T

2° Ab: Cytiva NA934V HRP αRabbit
1:5000 in 5% milk, 0.1% TBS-T

Exposure time: 2 min

NOTE: X = lane(s) not part of final figure

Figure 4B
Panel 3
P-MAPK

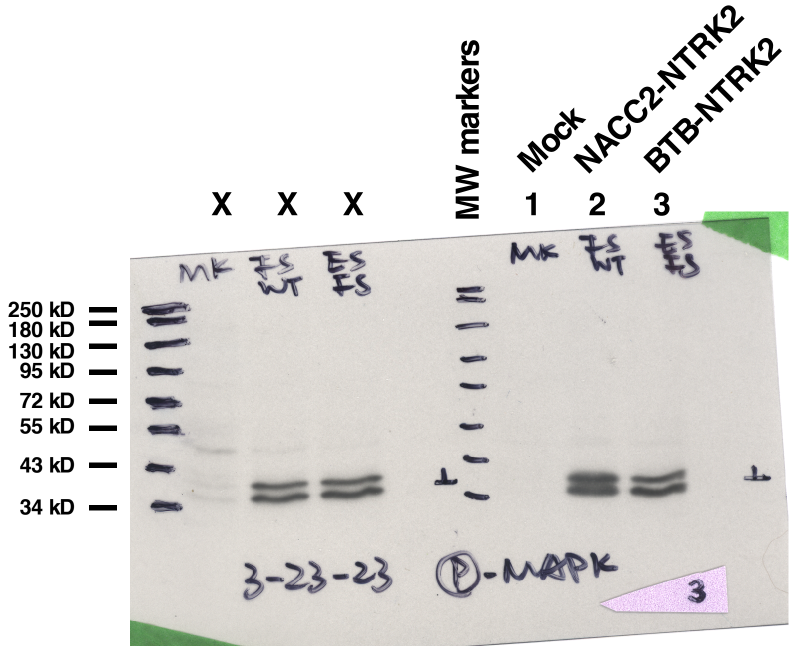

Date of immunoblot: 3/23/2023

12.5% SDS-PAGE, 180 V
40 µg lysate per lane

1° Ab: Cell Signaling #4370
Phospho-p44/42 MAPK (Erk1/2)
Rabbit mAb (Thr202/Tyr204)
(D13.14.4E)
1:2000 in 5% BSA, 0.1% TBS-T

2°Ab: Cytiva NA934V HRP αRabbit
1:2000 in 5% BSA, 0.1% TBS-T

Exposure time: 2 min

Figure 4B
Panel 4
MAPK

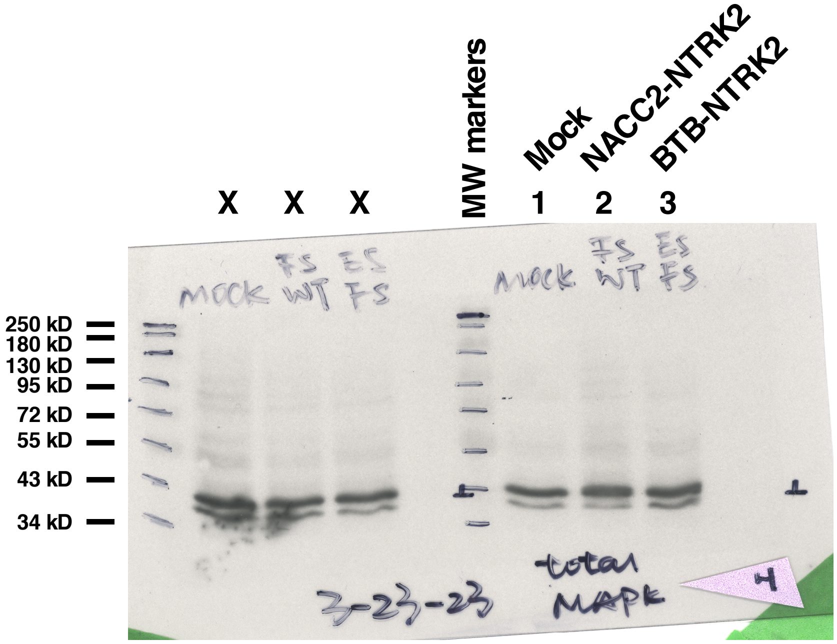

Date of immunoblot: 3/23/2023

12.5% SDS-PAGE, 180 V
40 µg lysate per lane

1° Ab: Cell Signaling #9102
p44/42 MAPK (Erk1/2)
Rabbit polyclonal Ab
1:2000 in 5% BSA, 0.1% TBS-T

2°Ab: Cytiva NA934V HRP αRabbit
1:2000 in 5% BSA, 0.1% TBS-T

Exposure time: 15 min

NOTE: X = lane(s) not part of final figure

Figure 4B
Panel 5
P-PLCγ1

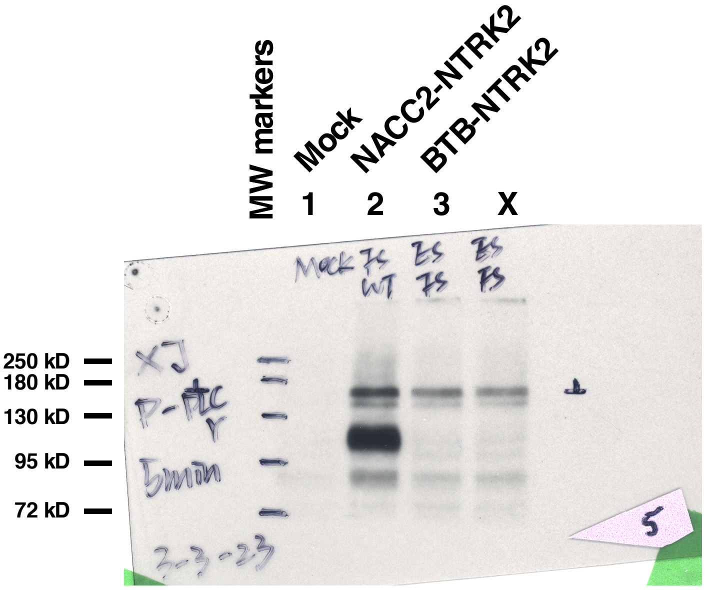

Date of immunoblot: 3/3/2023

10% SDS-PAGE, 180 V
40 µg lysate per lane

1° Ab: Cell Signaling #2821
Phospho-PLCγ1 (Tyr783) Antibody
Rabbit polyclonal AB
1:2000 in 5% BSA, 0.1% TBS-T

2°Ab: Cytiva NA934V HRP αRabbit
1:2000 in 5% BSA, 0.1% TBS-T

Exposure time: 5 min

Figure 4B
Panel 6
PLCγ1

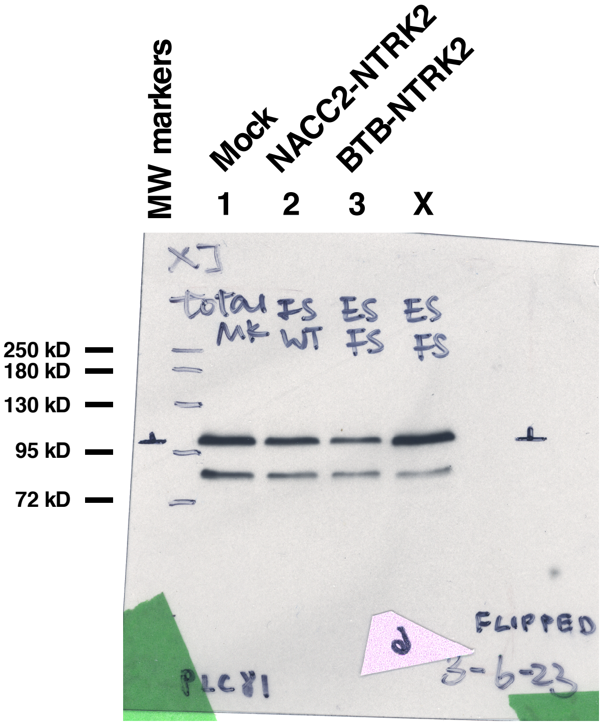

Date of immunoblot: 3/6/2023

10% SDS-PAGE, 180 V
40 µg lysate per lane

1° Ab: Santa Cruz (1249) sc-81
PLCγ1 Rabbit polyclonal Ab
1:1000 in 5% BSA, 0.1% TBS-T

2°Ab: Cytiva NA934V HRP αRabbit
1:2000 in 5% BSA, 0.1% TBS-T

Exposure time: 2 min

NOTE: X = lane(s) not part of final figure

Figure 4B
Panel 7
P-STAT3

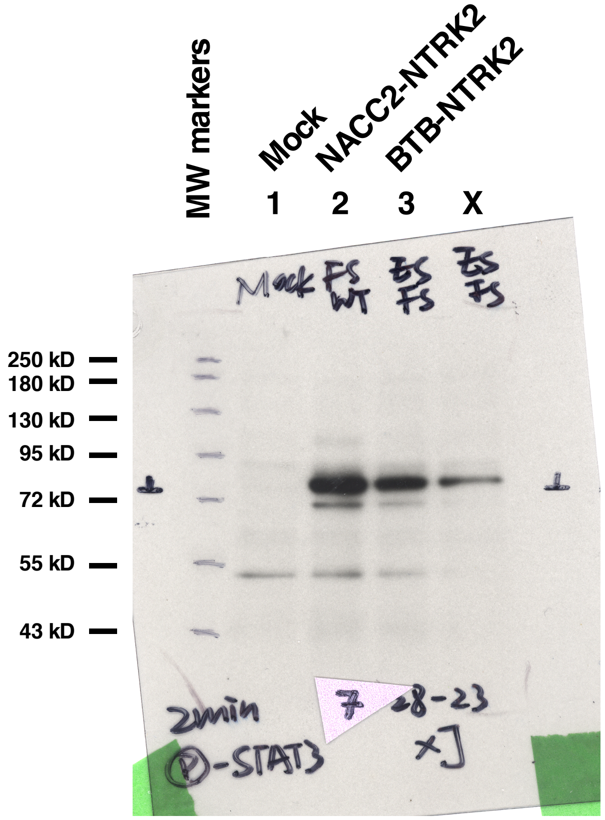

Date of immunoblot: 2/28/2023

10% SDS-PAGE, 180 V
40 µg lysate per lane

1° Ab: Cell Signaling #9145
Phospho-Stat3 (Tyr705) (D3A7)
Rabbit mAb
1:1000 in 5% BSA, 0.1% TBS-T

2°Ab: Cytiva NA934V HRP αRabbit
1:2000 in 5% BSA, 0.1% TBS-T

Exposure time: 2 min

Figure 4B
Panel 8
STAT3

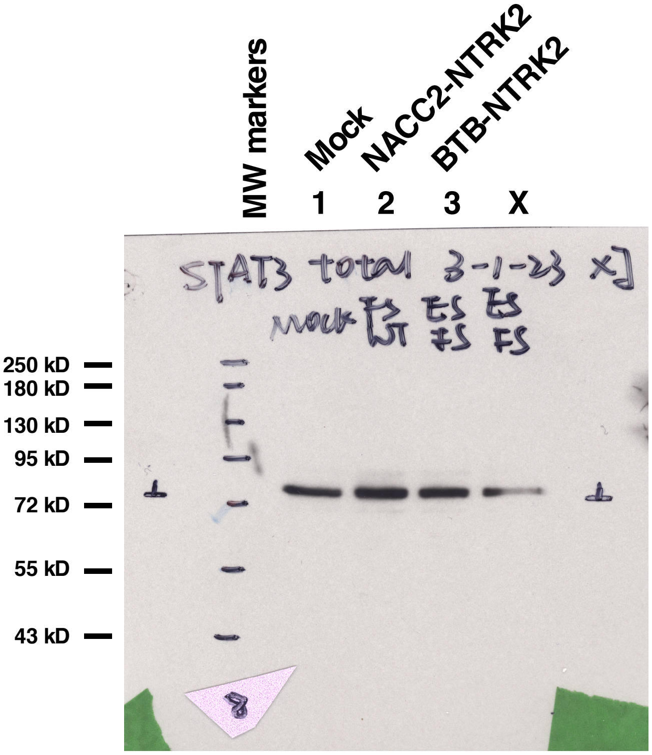

Date of immunoblot: 3/1/2023

10% SDS-PAGE, 180 V
40 µg lysate per lane

1° Ab: Cell Signaling #9139
Stat3 (124H6) Mouse mAb
1:500 in 5% BSA, 0.1% TBS-T

2°Ab: Cytiva NA931V HRP αMouse
1:2000 in 5% BSA, 0.1% TBS-T

Exposure time: 10 min

NOTE: **X = lane(s) not part of final figure**

Critical Domains for NACC2-NTRK2 Fusion Protein Activation

Authors: Wei Yang, April N. Meyer, Zian Jiang, Xuan Jiang, and Daniel J. Donoghue

Article in review for publication in PLoS One

Native Immunoblots for Figure 4D

Legend to Figure 4. NACC2 residues 121-418 provides potential covalent stabilization for NACC2-NTRK2 multimer.

(D) Lysates of HEK293T cells expressing NACC2-NTRK2 or BTB-NTRK2 were examined for homo-multimerization. Samples were loaded using non-reducing sample buffer (left panel), or reducing sample buffer containing β-mercaptoethanol (right panel), and then analyzed by SDS-PAGE and immunoblotted for NTRK2.

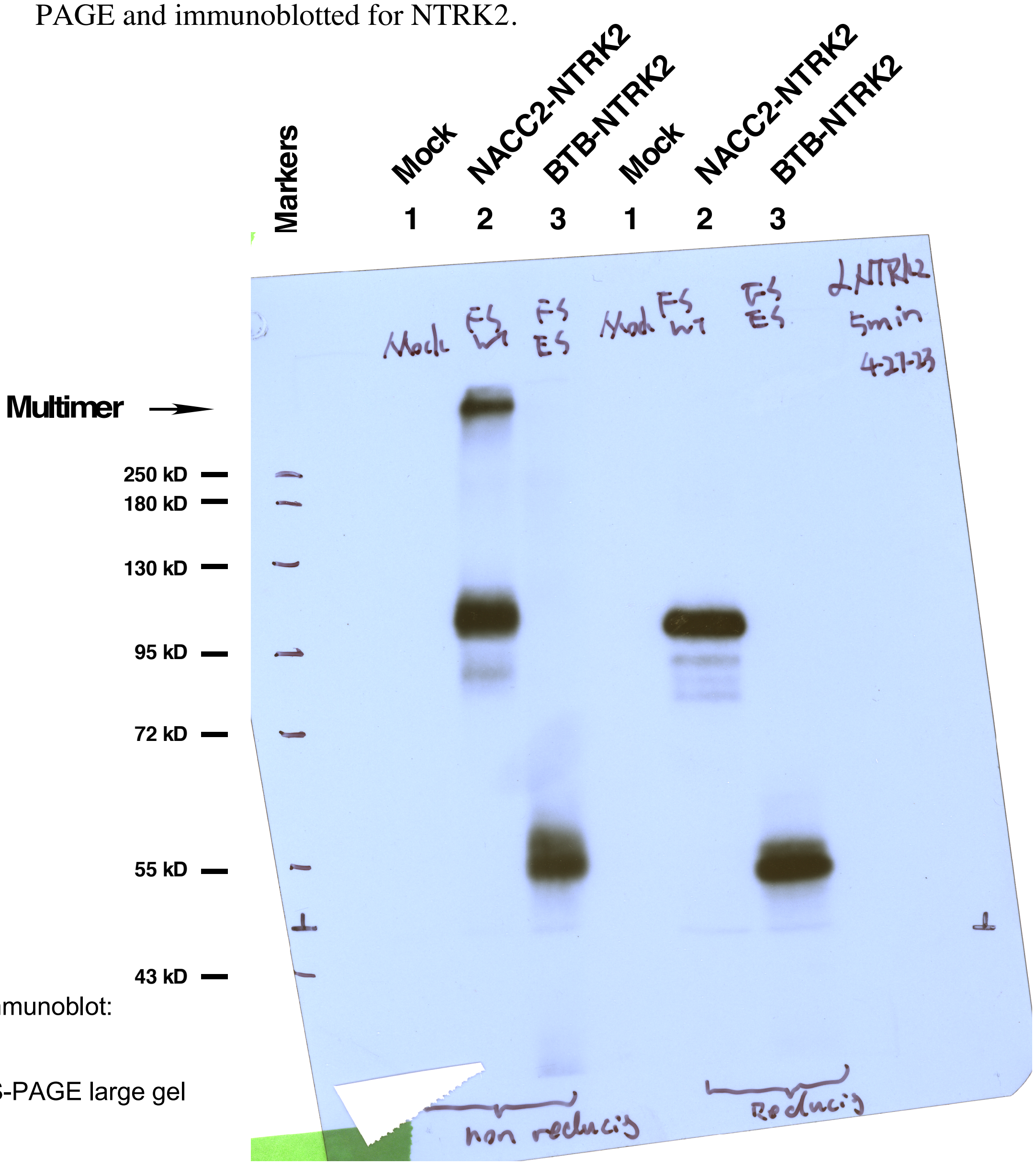

Date of immunoblot:
4/27/2023

7.5% SDS-PAGE large gel
50V O/N

1° Ab: Invitrogen PA5-86241
TrkB Rabbit polyclonal Ab
1:2000 in 5% BSA, 0.1% TBS-T

2° Ab: Cytiva NA934V HRP αRabbit
1:5000 in 5% milk, 0.1% TBS-T

Exposure time: 5 min

Figure 4D
Left Panel
IB: NTRK2
Non-reducing
Samples loaded in:
4% SDS,
10 mM NaPO4 pH7.0,
0.08% Bromophenol blue,
20% glycerol

Figure 4D
Right Panel
IB: NTRK2
Reducing
Samples loaded in:
2% SDS,
50mM Tris-Cl,
10% β-mercaptoethanol,
0.1% Bromophenol blue,
10% glycerol

Critical Domains for NACC2-NTRK2 Fusion Protein Activation

Authors: Wei Yang, April N. Meyer, Zian Jiang, Xuan Jiang, and Daniel J. Donoghue

Native Immunoblots for Figure 4E

Legend to Figure 4. NACC2 residues 121-418 provides potential covalent stabilization for NACC2-NTRK2 multimer.

(E) Hetero-multimerization assay of NACC2-NTRK2 and BTB-NTRK2. Fusion clones were cotransfected with NACC2 (lanes 4 and 5) and examined for their ability to bind to NACC2 in an immune complex prepared using antibodies against NTRK2. The 3rd panel shows clearly the binding of NACC2 to NACC2-NTRK2 (lane 4), indicated by a red arrowhead. NACC2 also clearly binds to BTB-NTRK2 (lane 5), again indicated by a red arrowhead. Control lysate blots are shown for NTRK2 (1st panel) and NACC2 (2nd panel). Control IP immunoblot is shown in 4th panel for NTRK2.

Native Immunoblots for Figure 4E are presented on the following page

Figure 4E
Top Panel
IB: NTRK2

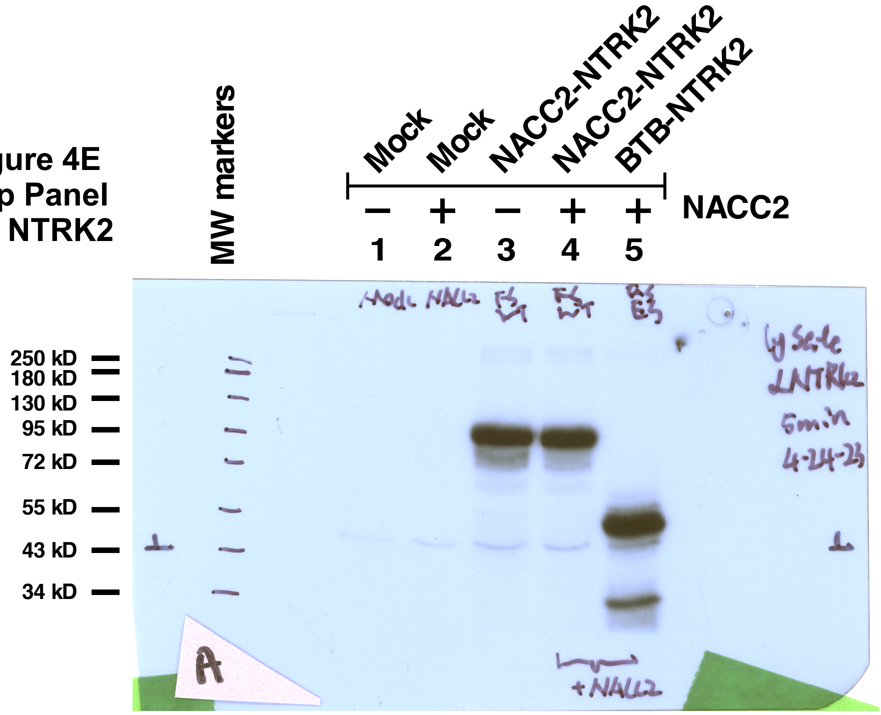

Date of immunoblot:4/24/2023

10% SDS-PAGE, 180 V
40 µg lysate per lane

1° Ab: Invitrogen #PA5-86241
TrkB Rabbit Polyclonal Ab
1:2000 in 5% BSA, 0.1% TBS-T

2° Ab: Cytiva NA934V HRP αRabbit
1:5000 in 5% milk, 0.1% TBS-T

Exposure time: 5 min

Figure 4E
2nd Panel
IB: NACC2

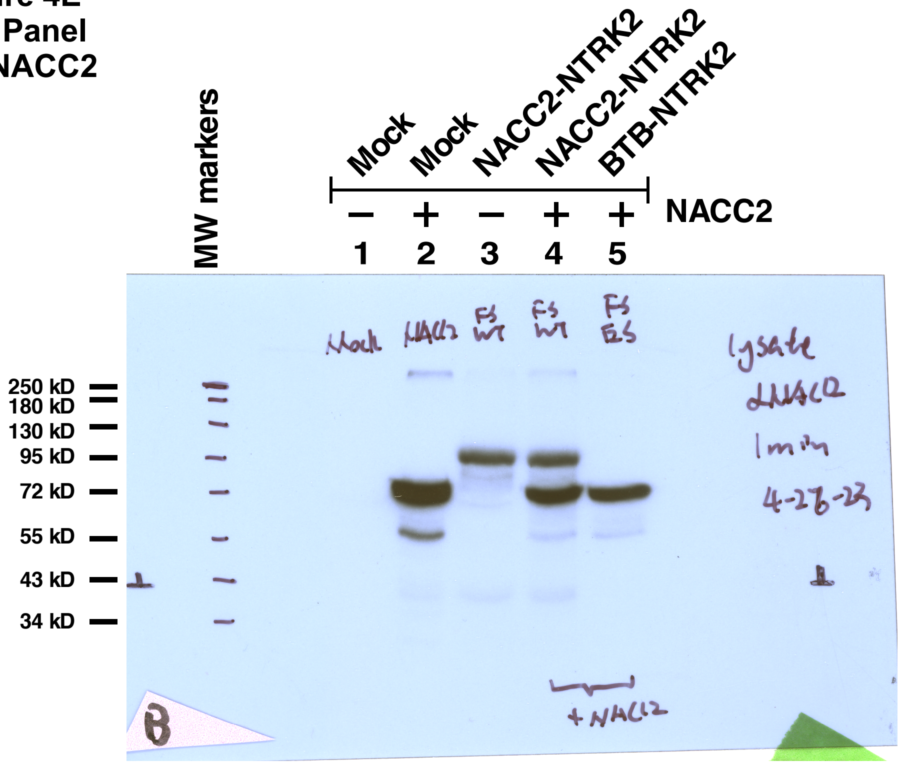

Date of immunoblot: 4/26/2023

10% SDS-PAGE, 180 V
40 µg lysate per lane

1° Ab: Bethyl A304-991A
Rabbit anti-NACC2/BTBD14A
Polyclonal Ab
1:2000 in 5% BSA, 0.1% TBS-T

2° Ab: Cytiva NA934V HRP αRabbit
1:5000 in 0.5% milk, 0.1% TBS-T

Exposure time: 1 min

Figure 4E
3rd Panel
IP: NTRK2
IB: NACC2

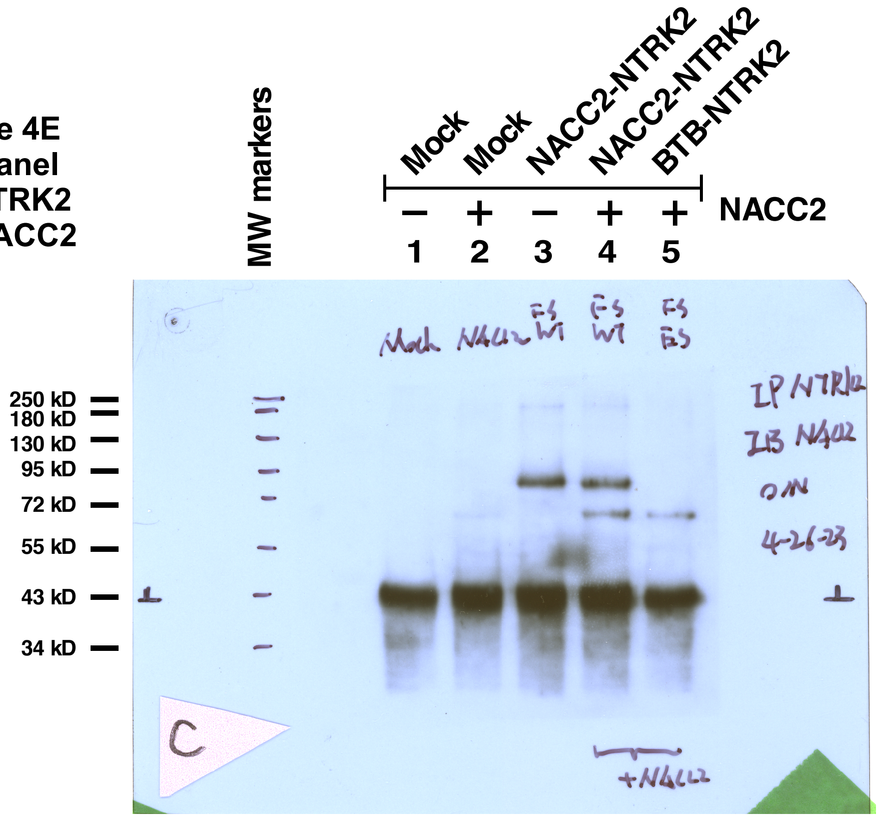

Date of immunoblot: 4/26/2023

10% SDS-PAGE, 180 V

IP Ab: Invitrogen #PA5-86241
TrkB Rabbit Polyclonal Ab
300µg total protein per sample IP'd
with 600ng of anti-TrkB antibody

1° Ab: Bethyl A304-991A
Rabbit anti-NACC2/BTBD14A
Polyclonal Ab
1:2000 in 5% BSA, 0.1% TBS-T

2° Ab: Cytiva NA934V HRP αRabbit
1:5000 in 0.5% milk, 0.1% TBS-T

Exposure time: O/N

Figure 4E
Bottom Panel
IP: NTRK2
IB: NTRK2

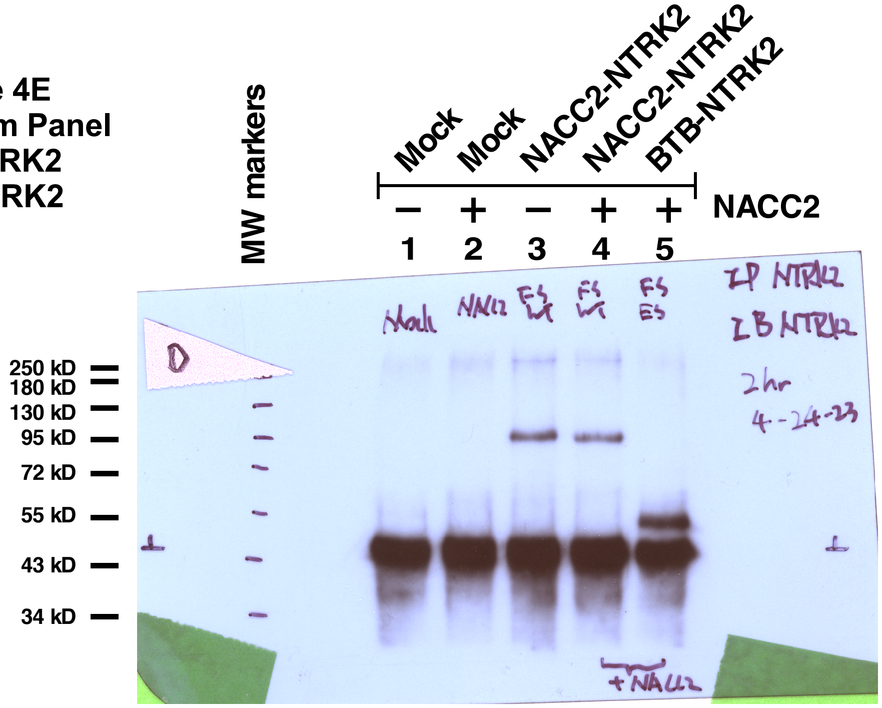

Date of immunoblot: 4/24/2023

10% SDS-PAGE, 180 V

IP Ab: Invitrogen #PA5-86241
TrkB Rabbit Polyclonal Ab
300µg total protein per sample IP'd
with 600ng of anti-TrkB antibody

1° Ab: Invitrogen #PA5-86241
TrkB Rabbit Polyclonal Ab
1:2000 in 5% BSA, 0.1% TBS-T

2° Ab: Cytiva NA934V HRP αRabbit
1:5000 in 0.5% milk, 0.1% TBS-T

Exposure time: 2 hr

Critical Domains for NACC2-NTRK2 Fusion Protein Activation

Authors: Wei Yang, April N. Meyer, Zian Jiang, Xuan Jiang, and Daniel J. Donoghue

Article in review for publication in PLoS One

Native Immunoblots for Figure 5B

Legend to Figure 5. Deletion of the transmembrane helix promotes greater stability and biological activity.

(B) HEK293T cells expressing NACC2-NTRK2, NACC2-NTRK2-ΔTM and NACC2-NTRK2(ex4:ex15) were examined for activation of downstream signaling pathways, as described previously for Figures 1D and 3C.

Native Immunoblots for Figure 5B are presented on the following pages

Figure 5B
Panel 1
P-NTRK2

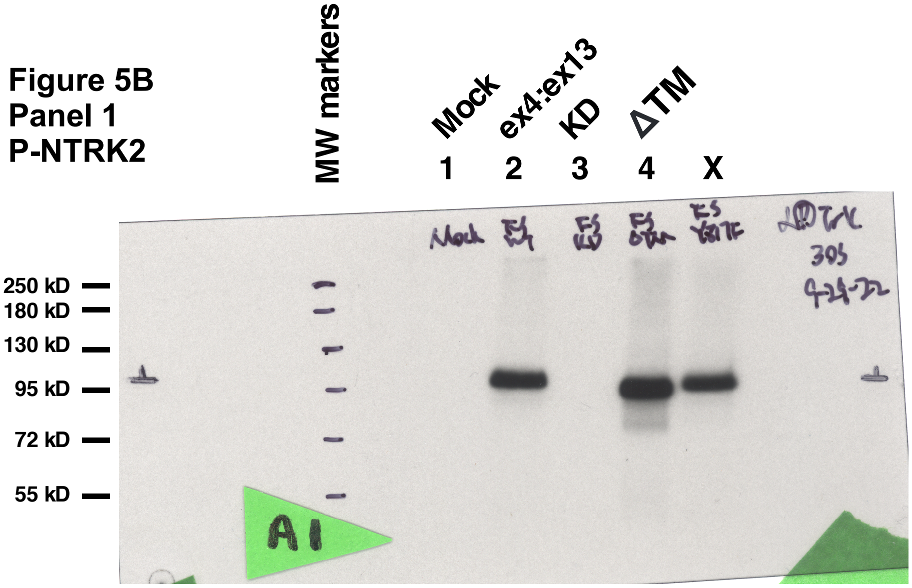

Date of immunoblot: 9/25/2022

10% SDS-PAGE, 180 V
40 µg lysate per lane

1° Ab: Cell Signaling #4621
Phospho-TrkA/TrkB Rabbit mAb
1:2000 in 5% BSA, 0.1% TBS-T

2° Ab: Cytiva NA934V HRP αRabbit
1:5000 in 5% BSA, 0.1% TBS-T

Exposure time: 30 sec

Figure 5B
Panel 2
NTRK2

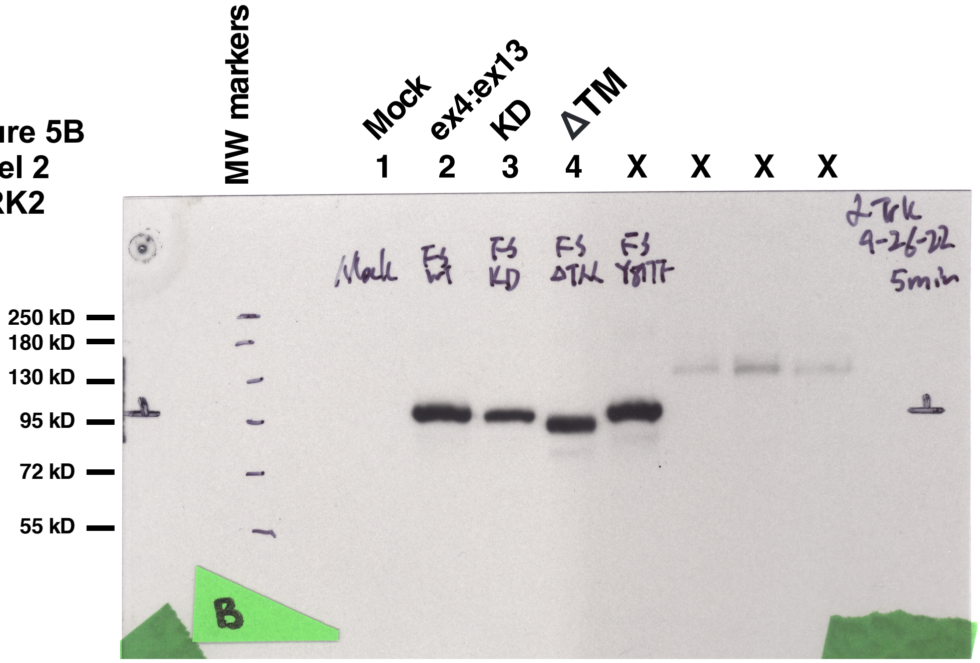

Date of immunoblot: 9/26/2022

10% SDS-PAGE, 180 V
40 µg lysate per lane

1° Ab: Invitrogen PA5-86241
TrkB Rabbit polyclonal Ab
1:2000 in 5% BSA, 0.1% TBS-T

2° Ab: Cytiva NA934V HRP αRabbit
1:5000 in 5% milk, 0.1% TBS-T

Exposure time: 5 min

NOTE: X = lane(s) not part of final figure

Figure 5B
Panel 3
P-MAPK

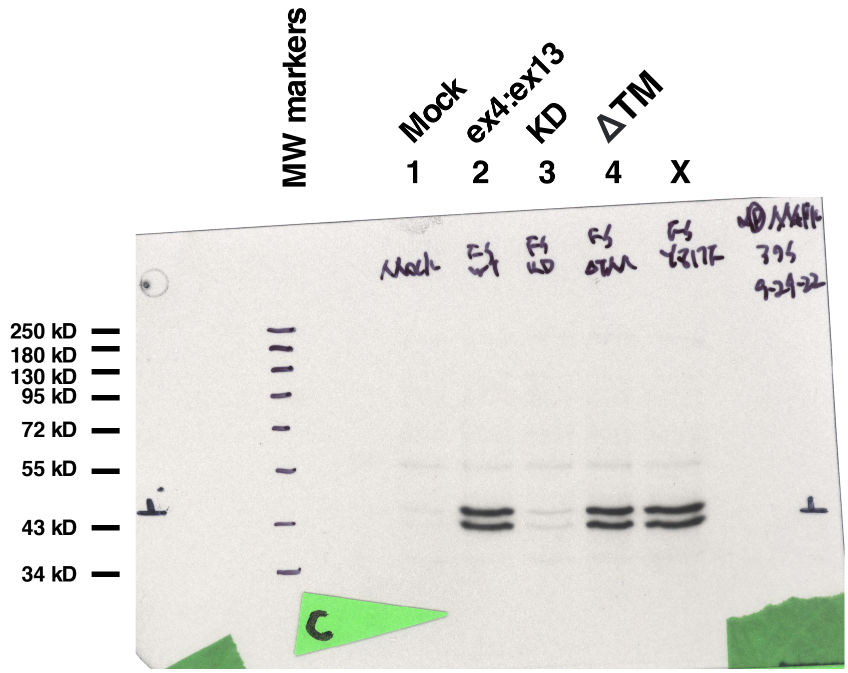

Date of immunoblot: 9/24/2022

12.5% SDS-PAGE, 180 V
40 µg lysate per lane

1° Ab: Cell Signaling #4370
Phospho-p44/42 MAPK (Erk1/2)
Rabbit mAb (Thr202/Tyr204)
(D13.14.4E)
1:2000 in 5% BSA, 0.1% TBS-T

2°Ab: Cytiva NA934V HRP αRabbit
1:2000 in 5% BSA, 0.1% TBS-T

Exposure time: 39 sec

Figure 5B
Panel 4
MAPK

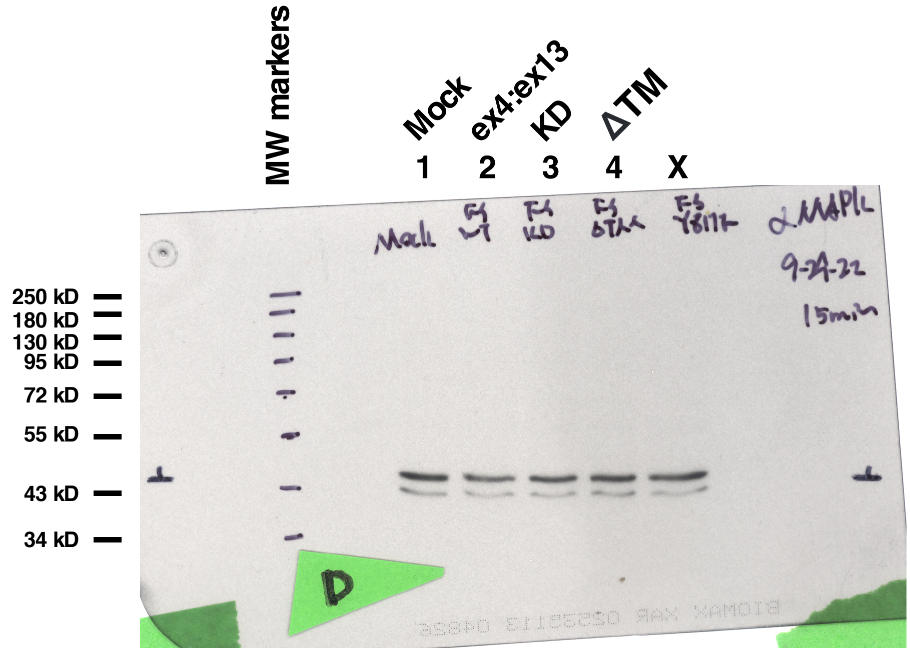

Date of immunoblot: 9/24/2022

12.5% SDS-PAGE, 180 V
40 µg lysate per lane

1° Ab: Cell Signaling #9102
p44/42 MAPK (Erk1/2)
Rabbit polyclonal Ab
1:2000 in 5% BSA, 0.1% TBS-T

2°Ab: Cytiva NA934V HRP αRabbit
1:2000 in 5% BSA, 0.1% TBS-T

Exposure time: 15 min

NOTE: X = lane(s) not part of final figure

Figure 5B
Panel 5
P-PLCγ1

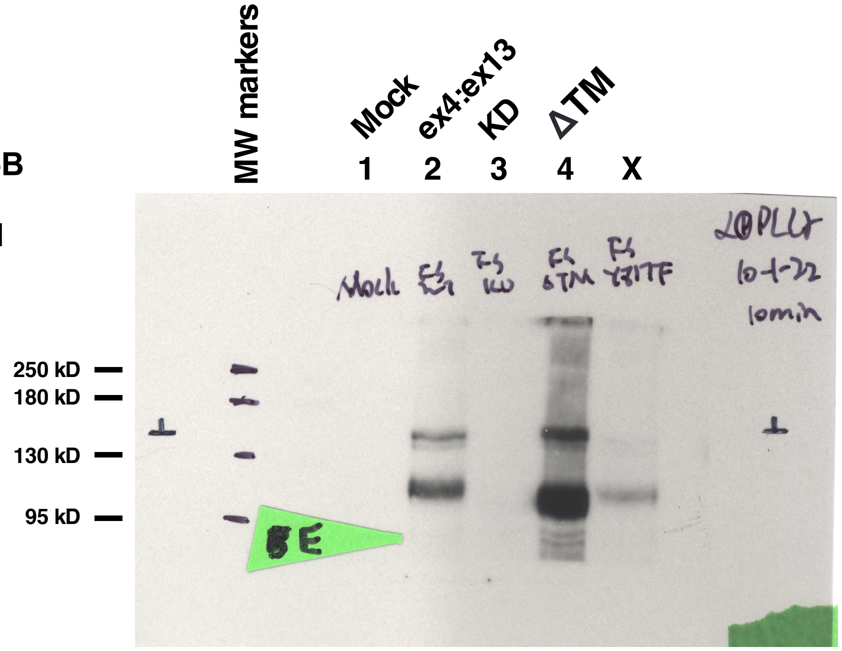

Date of immunoblot: 10/1/2022

10% SDS-PAGE, 180 V
40 µg lysate per lane

1° Ab: Cell Signaling #2821
Phospho-PLCγ1 (Tyr783) Antibody
Rabbit polyclonal AB
1:2000 in 5% BSA, 0.1% TBS-T

2°Ab: Cytiva NA934V HRP αRabbit
1:2000 in 5% BSA, 0.1% TBS-T

Exposure time: 10 min

Figure 5B
Panel 6
PLCγ1

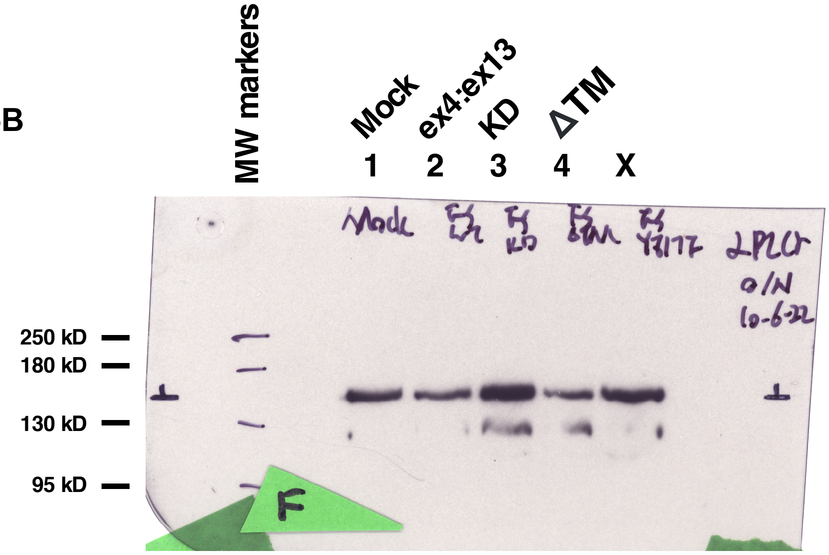

Date of immunoblot: 10/6/2022

10% SDS-PAGE, 180 V
40 µg lysate per lane

1° Ab: Santa Cruz (1249) sc-81
PLCγ1 Rabbit polyclonal Ab
1:1000 in 5% BSA, 0.1% TBS-T

2°Ab: Cytiva NA934V HRP αRabbit
1:2000 in 5% BSA, 0.1% TBS-T

Exposure time: O/N

NOTE: X = lane(s) not part of final figure

Figure 5B
Panel 7
P-STAT3

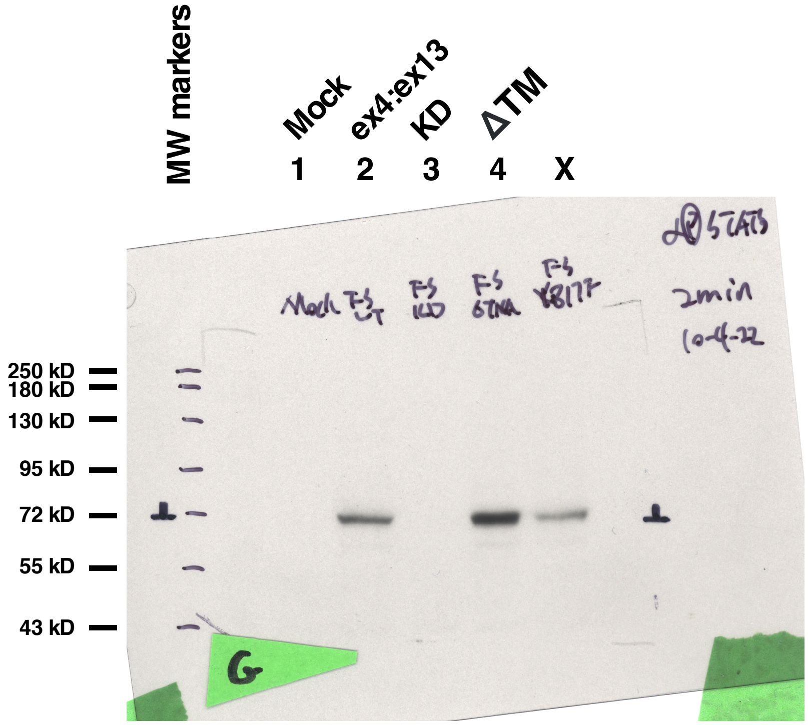

Date of immunoblot: 10/4/2022

10% SDS-PAGE, 180 V
40 µg lysate per lane

1° Ab: Cell Signaling #9145
Phospho-Stat3 (Tyr705) (D3A7)
Rabbit mAb
1:1000 in 5% BSA, 0.1% TBS-T

2°Ab: Cytiva NA934V HRP αRabbit
1:2000 in 5% BSA, 0.1% TBS-T

Exposure time: 2 min

Figure 5B
Panel 8
STAT3

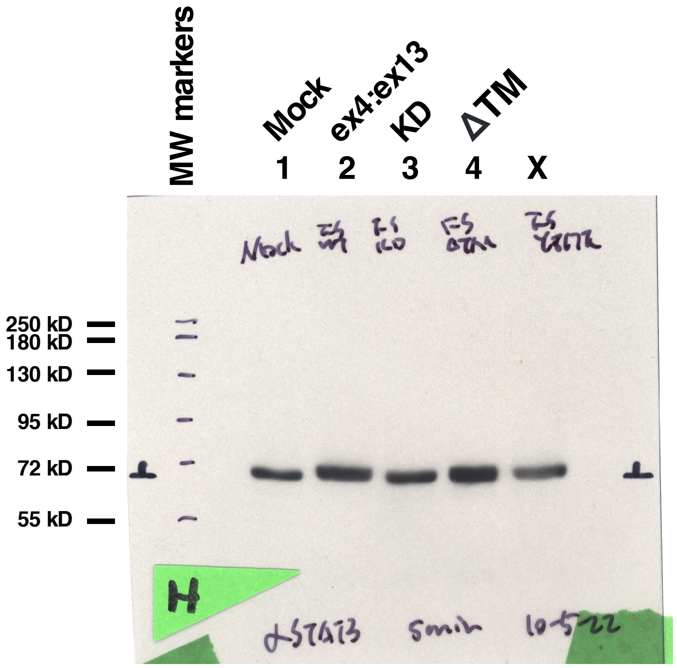

Date of immunoblot: 10/5/2022

10% SDS-PAGE, 180 V
40 µg lysate per lane

1° Ab: Cell Signaling #9139
Stat3 (124H6) Mouse mAb
1:500 in 5% BSA, 0.1% TBS-T

2°Ab: Cytiva NA931V HRP αMouse
1:2000 in 5% BSA, 0.1% TBS-T

Exposure time: 5 min

NOTE: X = lane(s) not part of final figure

Critical Domains for NACC2-NTRK2 Fusion Protein Activation

Authors: Wei Yang, April N. Meyer, Zian Jiang, Xuan Jiang, and Daniel J. Donoghue

Article in review for publication in PLoS One

Native Immunoblots for Figure 5E

Legend to Figure 5. Deletion of the transmembrane helix promotes greater stability and biological activity.

(E) Lysates prepared from cells expressing either NACC2-NTRK2 (left lanes) or NACC2-NTRK2-ΔTM (right lanes) and treated with cycloheximide for the indicated time points were examined by SDS-PAGE and immunoblotted for NTRK2 The left lanes show decreasing amounts of NACC2-NTRK2 in the presence of cycloheximide, while the right lanes reveal little or no change in NACC2-NTRK2-ΔTM under identical conditions.

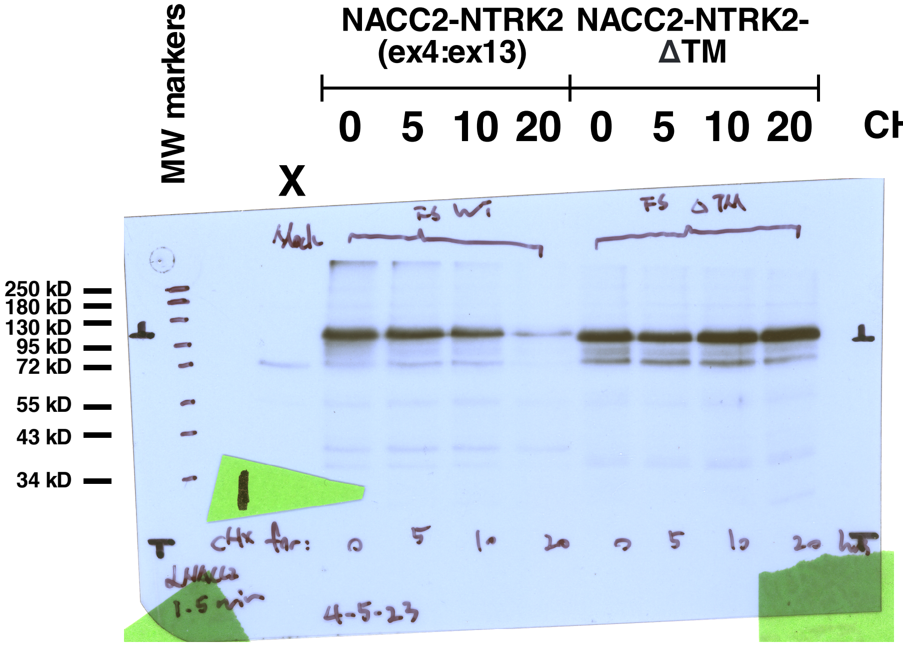

Date of immunoblot: 4/5/2023

10% SDS-PAGE, 180 V
40 µg lysate per lane

1° Ab: Bethyl A304-991A
Rabbit anti-NACC2/BTBD14A
Polyclonal Ab
1:2000 in 5% BSA, 0.1% TBS-T

2° Ab: Cytiva NA934V HRP αRabbit
1:5000 in 0.5% milk, 0.1% TBS-T

Exposure time: 5 min

NOTE: X = lane not part of final figure
